# Neurobehavioral Impairment in Pediatric Brain Tumor Survivors: A Meta-Analysis

**DOI:** 10.3390/cancers14133269

**Published:** 2022-07-04

**Authors:** Yuliang Wang, Anthony Pak Yin Liu, Tatia Mei-Chun Lee, Wilfred Hing Sang Wong, Daniel Yee Tak Fong, Lok Kan Leung, Matthew Ming Kong Shing, Dennis Tak-Loi Ku, Godfrey Chi-Fung Chan, Winnie Wan-Yee Tso

**Affiliations:** 1 Department of Paediatrics and Adolescent Medicine, The University of Hong Kong, Hong Kong, China; u3007730@connect.hku.hk (Y.W.); apyliu@hku.hk (A.P.Y.L.); whswong@hku.hk (W.H.S.W.); oscarlk@hku.hk (L.K.L.); 2 Department of Paediatrics and Adolescent Medicine, Hong Kong Children’s Hospital, Hong Kong, China; mk-shing@cuhk.edu.hk; 3 State Key Laboratory of Brain and Cognitive Sciences, The University of Hong Kong, Hong Kong, China; tmclee@hku.hk (T.M.-C.L.); dennisku@hotmail.com (D.T.-L.K.); 4 Laboratory of Neuropsychology and Human Neuroscience, Department of Psychology, The University of Hong Kong, Hong Kong, China; 5 School of Nursing, The University of Hong Kong, Hong Kong, China; dytfong@hku.hk

**Keywords:** pediatric brain tumor survivors, neurobehavioral impairment, meta-analysis

## Abstract

**Simple Summary:**

Through synthesizing studies regarding neurobehavioral impairment of pediatric brain tumor survivors (PBTS) in the past decade, this meta-analysis found that PBTS are at higher risk of attention problems, emotional difficulties and psychosocial problems compared to the healthy population. Future studies should focus on exploring potential interventions for PBTS at risk of neurobehavioral impairment to improve the long-term psychological outcomes.

**Abstract:**

Purpose: The neurocognitive outcomes of pediatric brain tumor survivors have been extensively studied but the risk and predictors for neurobehavioral impairment are less clearly defined. We systematically analyzed the rates of emotional, psychosocial, and attention problems in pediatric brain tumor survivors. Methods: PubMed, Web of Science, Embase, Scopus, and Cochrane were searched for articles published between January 2012 to April 2022. Eligible studies reported neurobehavioral outcomes for PBTS aged 2 to <23 years with a brain tumor diagnosis before 18 years of age. A random-effect meta-analysis was performed in R. Results: The search yielded 1187 unique publications, of which 50 were included in the quantitative analysis. The estimated risk of having emotional, psychosocial, and attention problems were 15% (95%CI 10–20%), 12% (95%CI 9–16%), and 12% (95%CI 9–16%), respectively. PBTS were more likely to have emotional difficulties (Hedge’s g = 0.43 [95%CI 0.34–0.52]), psychosocial problems (Hedge’s g = 0.46 [95%CI 0.33–0.58]), and attention problems (Hedge’s g = 0.48 [95%CI 0.34–0.63]) compared to normal/healthy control subjects. There was no significant difference in the rates of neurobehavioral impairment between children with and without history of cranial radiotherapy. Conclusions: PBTS are at elevated risk of neurobehavioral impairment. Neurobehavioral monitoring should be considered as the standard of care for PBTS.

## 1. Introduction

Brain tumors are the most common solid tumors affecting children and adolescents, accounting for approximately 27% of pediatric cancers and affecting approximately 3000 children per year in the United States [1]. The prognosis of pediatric malignancies has improved dramatically over the past decades, with 70% of children diagnosed with cancers in developed countries surviving their illness [2]. Because of the improved survival, there are new challenges in the long-term management of childhood cancer survivors, who often require multi-disciplinary care, particularly for their medical and psychosocial sequalae that might adversely impact their quality of life [3]. Moreover, pediatric brain tumor survivors (PBTS) typically also have neurocognitive and behavioral problems [4,5,6,7,8,9]. Given that cranial radiotherapy (RT) is associated with a high risk of neurotoxicity in PBTS, many survivorship studies have focused on the cognitive outcomes of PBTS [8,9,10,11,12,13,14], but very few studies have investigated the neurobehavioral outcomes.

Neurobehavioral disorders are very common in children and adolescents, affecting 4.4–9.8% of the general pediatric population [15]. However, children with acquired brain injury [16] that can significantly affect the developing brain such as PBTS have a much higher risk of neurobehavioral disorders. Earlier research on the neurobehavioral outcomes of PBTS showed that the majority of PBTS did not exhibit clinically significant psychopathology [17]. However, recent studies have shown that PBTS are more prone to emotional and behavioral difficulties and poorer psychosocial well-being that necessitate psychiatric support and rehabilitation services [18,19,20,21]. Besides emotional problems, studies also showed that PBTS are at risk of internalizing problems such as anxiety and depression [22], whereas a small number of PBTS might also exhibit externalizing behaviours [23]. Moreover, PBTS were also more likely to have fewer friendships, with more social problems and social isolation, and display less leadership compared to their peers and children with other cancers [19,20,21]. In addition, adolescents and young adult survivors often have weaksocial skills and experience difficulties in pursuing education and employment [18]. Another common late effect seen in PBTS are attention problems that are often associated with psychosocial and academic difficulties [24]. Earlier studies showed conflicting findings on neurobehavioral outcomes in PBTS, which might be due to the different tumor types or treatment modalities across these studies [25]. Some studies found that PBTS with a history of cranial irradiation or intrathecal chemotherapy had a higher risk of behavioral and emotional problems [23,26,27]. Despite the importance of neurobehavioral function in PTBS, it remains unclear whether PBTS have a higher risk of neurobehavioral problems compared to healthy children, particularly specific neurobehavioral impairments that affect emotional and psychosocial functioning.

Given the increased survival in PBTS, there needs to be more efforts to understand and improve the long-term outcomes [28], particularly the risk of neurobehavioral impairments such as emotional, psychosocial, and attention problems. Such information will be useful to ensure that PBTS at risk of neurobehavioral impairment can receive early diagnosis and interventions. This study aims to investigate the rates of emotional, psychosocial, and attention problems based on survivor-reported or proxy-reported outcomes using validated assessment scales. As PBTS might not have received detailed neuropsychological assessment at follow-up, survivor-reported or proxy-reported questionnaires might serve as good screening tools for PBTS at risk of neurobehavioral problems.

This meta-analysis was conducted to examine the risk of emotional, psychosocial, and attention problems in PBTS by focusing on studies in the past decade. The study also aimed to identify the risk factors pre-disposing PBTS to poorer neurobehavioral outcomes.

## 2. Materials and Methods

### 2.1. Searching Strategy

We searched PubMed, Scopus Embase, Web of Science, and Cochrane in April 2022 for articles published from 1 January 2012 to 20 April 2022. The following string was used to search the databases [“CNS tumor” OR “brain tumor” OR “brain Oncology” or neuro-oncology OR medulloblastomas OR “pilocytic astrocytoma” OR craniopharyngiomas OR “germ cell tumors” OR glioma OR ependymal OR glioneuronal OR embryonal in Title Abstract Keyword] AND [children OR pediatric OR adolescent OR toddler OR preschool OR teen OR teenager OR childhood in Title Abstract Keyword] AND [“social difficulties” OR “social outcome” OR “social problems” OR “social deficits” OR “emotional difficulties” OR “emotional problems” OR “attention deficits” OR “attention problems” OR ADHD OR “attention deficit hyperactivity disorder” OR “autism spectrum disorder” OR autism OR “developmental outcomes” OR “behavioral difficulties” OR neurobehavior OR neuropsychological OR psychiatric OR psychosocial OR depression OR anxiety OR internalizing OR externalizing in Title Abstract Keyword]. Word variations were also searched. References from the identified studies and relevant reviews were also retrieved and searched. See File S1 for the specific search strings used in each database.

### 2.2. Study Selection

Assessed articles are screened by two independent reviewers according to the following inclusion and exclusion criteria:

#### 2.2.1. Inclusion Criteria

The inclusion criteria were as follows: (1) participants diagnosed with brain tumor before the age of 18 years; (2) assessed participants between the age of two and 23; (3) assessed at least one of the three aspects of neurobehavioral impairment by validated standard scales: (a) autistic features/psychosocial problems/psychosocial outcomes; (b) emotional problems/internalizing problem/externalizing problem; (c) attention deficits/attention problems; (4) reported original research data; and (5) studies published in English.

#### 2.2.2. Exclusion Criteria

The exclusion criteria were as follows: (1) case study, conference abstract and papers; (2) no validated standard scales measuring neurobehavioral impairment; (3) norm/clinical cut-off or healthy control scores were not provided for the scale; (4) data not retrievable for calculating either the absolute risk or the standard mean difference (compared to the population norm or healthy control) of the psychosocial/emotion/attention problems in PBT participants; (5) researched on paediatric cancer survivor cohort while CNS paediatric cancer survivor’s data are not provided separately; (6) assessed overall psychological/neurobehavioral impairment while psychosocial/emotion/attention scores are not provided separately.

#### 2.2.3. Selection Procedure

Titles and abstracts of assessed papers were first screened by the two reviewers (YW and WWYT) for potentially eligible studies. Those identified studies were then reviewed in full text. In each step, disagreement was solved through consensus by the two reviewers. The inter-rater reliability is calculated in the inclusion process.

### 2.3. Data Extraction and Quality Assessment

Data (mean, standard deviation, sample size, clinical cut-offs, etc.) required to calculate the standard mean difference and absolute risk for neurobehavioral impairments in PBTS were retrieved from each study. The assessment results at baseline and at all follow-up time points were also retrieved from the studies. For studies containing more than one independent cohort, the data of these cohorts were recorded separately. For studies reporting more than one measurement in one aspect of the neurobehavioral impairment (psychosocial/emotional/attention), the pooled standard mean difference was calculated [29]. The risk of methodological bias in each study was rated by the three independent reviewers (YW, LKL and WWYT) according to the STROBE checklist (method section) for observational studies [30]. The overall risk of bias was rated as ‘low’, ‘medium’ and ‘high’. Discrepancies in the ratings were resolved by consensus.

### 2.4. Statistical Methods

A meta-analysis was conducted to synthesize the findings on the risk of neurobehavioral problems in PBTS in the following two aspects: (1) the absolute risk: the proportion of PBTS who were below the clinical cut-offs for psychosocial, emotional, and attention problems from each identified study; and (2) the standard mean difference: the psychosocial, emotional, and attention problems in PBTS compared to the population norm and healthy controls. A random-effect model was used to pool the results from the different studies. The standard mean difference was measured by Hedge’s g. The heterogeneity across studies was evaluated by I^2^ statistics, with I^2^ ≥ 50% indicating substantial heterogeneity; and the significance of heterogeneity was examined by an χ^2^ test. For pooling the absolute risk and standard mean differences, self-reported data was used for children aged 12 and above, whereas parent-reported data was used for children below the age of 12. Subgroup analysis was conducted to examine categorical moderating factors, including reporting methods (self-report, parent-report, and teacher-report), comparison groups (healthy control vs. population norm), and treatments (with or without a history of radio therapy). Peters’ Regression Test [31] and Egger’s test [32] were used to determine the publication bias in binary meta-analytical outcomes (absolute risk) and standard mean differences (Hedges’ g), respectively. Meta-regression was used to examine moderating factors, including age at assessment, age at diagnosis, and follow-up time. A *p*-value < 0.05 was considered to be statistically significant. All analyses were conducted in R 4.1.1 using the ‘meta’ and ‘esc’ packages [33].

This meta-analysis follows the PRISMA guidelines [34] and is registered in PROSPERO (ID CRD42022328593).

## 3. Results

**Figure 1:**
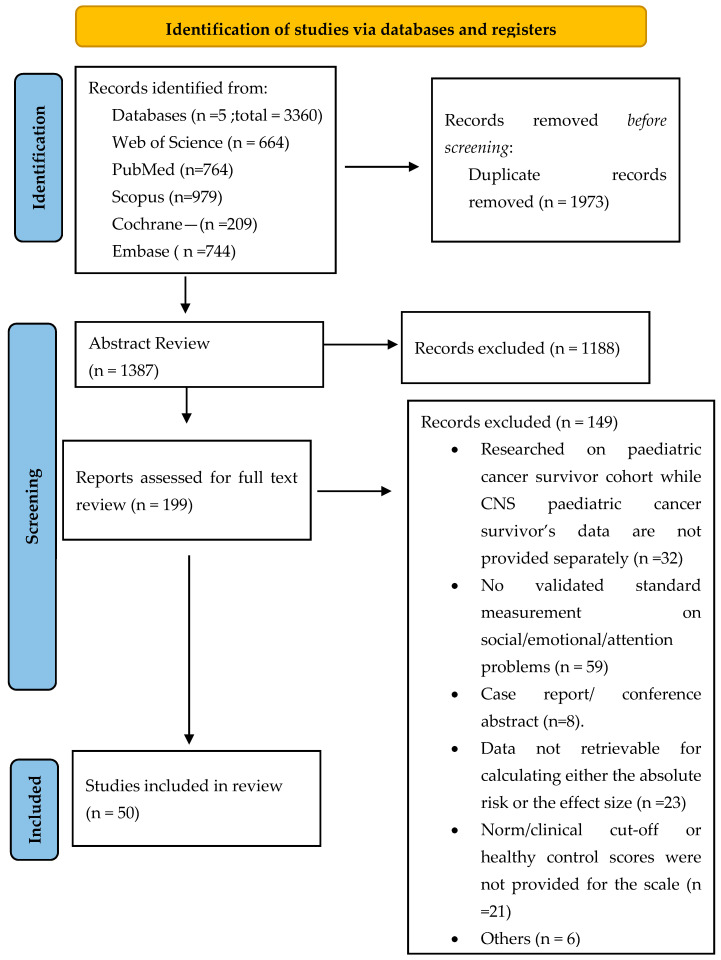
Inclusion of studies.

The database searches yielded 3360 results, of which 1387 unique publications were further reviewed, and 50 studies were included in the final meta-analysis (see Figure 1). The Cohen’s kappa for the inter-rater reliability of the two independent reviewers throughout the screening process was 0.82, indicating good agreement. Any disagreements in study eligibility were discussed and resolved by consensus.

### 3.1. Study Characteristics

Table 1 gives a summary of the characteristics of the included studies. Among the 50 included studies, 37 (74%) included a heterogeneous sample of PBTS, 13 (26%) included a cohort of children with a specific type of brain tumor, three (6%) included only participants were not treated with radio therapy (RT), 10 (20%) included only participants treated with RT, and 36 (72%) reported a heterogeneous sample of participants with or without RT treatment. Of the reported neurobehavioral measures, 36 studies reported psychosocial problems, 33 reported emotional difficulties, and 21 reported attention problems. The sample size of all included studies was 3581 PBTS, ranging from seven to 665 across individual studies. The mean age at diagnosis of brain tumor was 7.32 years (SD = 2.53) and mean age at assessment was 11.73 years (SD = 3.69).

### 3.2. Absolute Risk of Neurobehavioral Problems in PBTS

#### 3.2.1. Absolute Risk—Attention Problems

**Figure 2:**
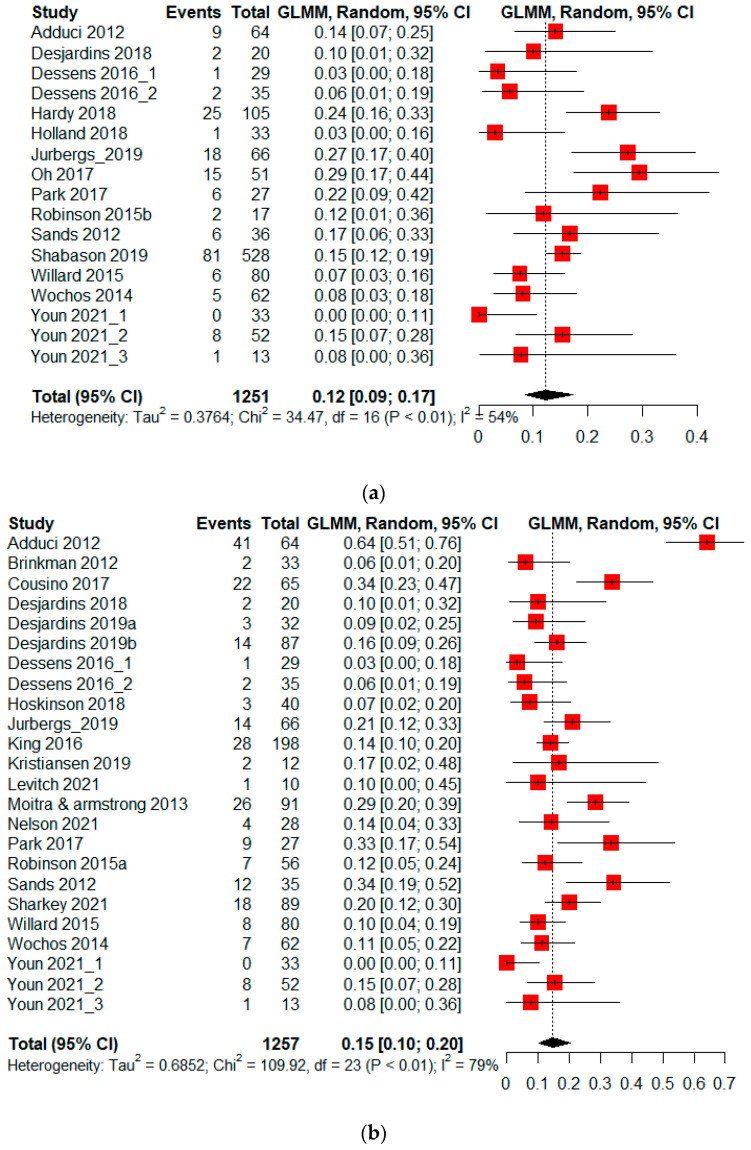

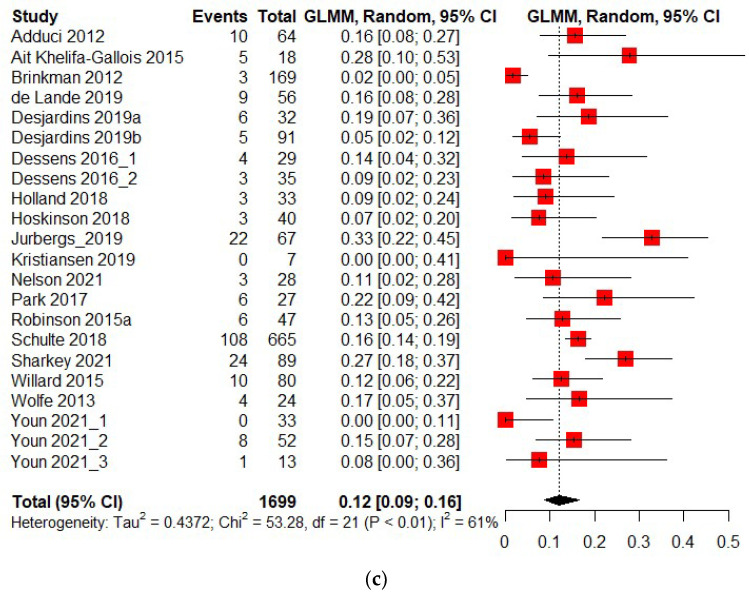
(**a**–**c**) Absolute risk of pediatric brain tumor survivors having neurobehavioral impairment. Dessens et al. (2016) [49] and Youn et al. (2021) [83] reported more than one independent cohort in their study [24,30,36,37,39,40,42,43,45,46,47,49,55,56,57,58,60,61,63,64,65,66,70,71,72,73,74,75,77,81,82,83]. (**a**) Abso-lute risk—attention problems; (**b**) Absolute risk—emotional; (**c**) Absolute risk—psychosocial problems.

The proportion of PBTS whose attention problems were below the clinical cut-off was reported in 14 studies (*n* = 1251) (Figure 2a). The pooled absolute risk of PBTS having attention problems was 12% (95% CI 9–17%). There was a significant level of heterogeneity across the different studies (I^2^ = 54%, *p* < 0.01) and no significant publication bias was identified t (15) = 0.36, *p* = 0.72, Figure S5a.

#### 3.2.2. Absolute Risk—Emotional Difficulties

The proportion of PBTS whose emotional difficulties were below the clinical cut-off was reported in 21 studies (*n* = 1257) (Figure 2b). The pooled absolute risk of PBTS having emotional difficulties was 15% (95% CI 10–20%). There was a significant level of heterogeneity across the different studies (I^2^ = 79%, *p* < 0.01). No significant publication bias was observed t (22) = −0.47, *p* = 0.646 (Figure S5b).

#### 3.2.3. Absolute Risk—Psychosocial Problems

The proportion of PBTS whose psychosocial problems were below the clinical cut-off was reported in 19 studies (*n* = 1699) (Figure 2c). The pooled absolute risk of PBTS having psychosocial problems was 12% (95% CI 9–16%). There was a significant level of heterogeneity across the different studies (I2 = 61%, *p* < 0.01). Publication bias was not significant t (20) = 0.12, *p* = 0.908 (Figure S5c). 

### 3.3. The Standard Mean Difference of Neurobehavioral Impairment in PBTS Compared to the Population Norm or Healthy Control

#### 3.3.1. Standard Mean Difference—Attention Problems

**Figure 3:**
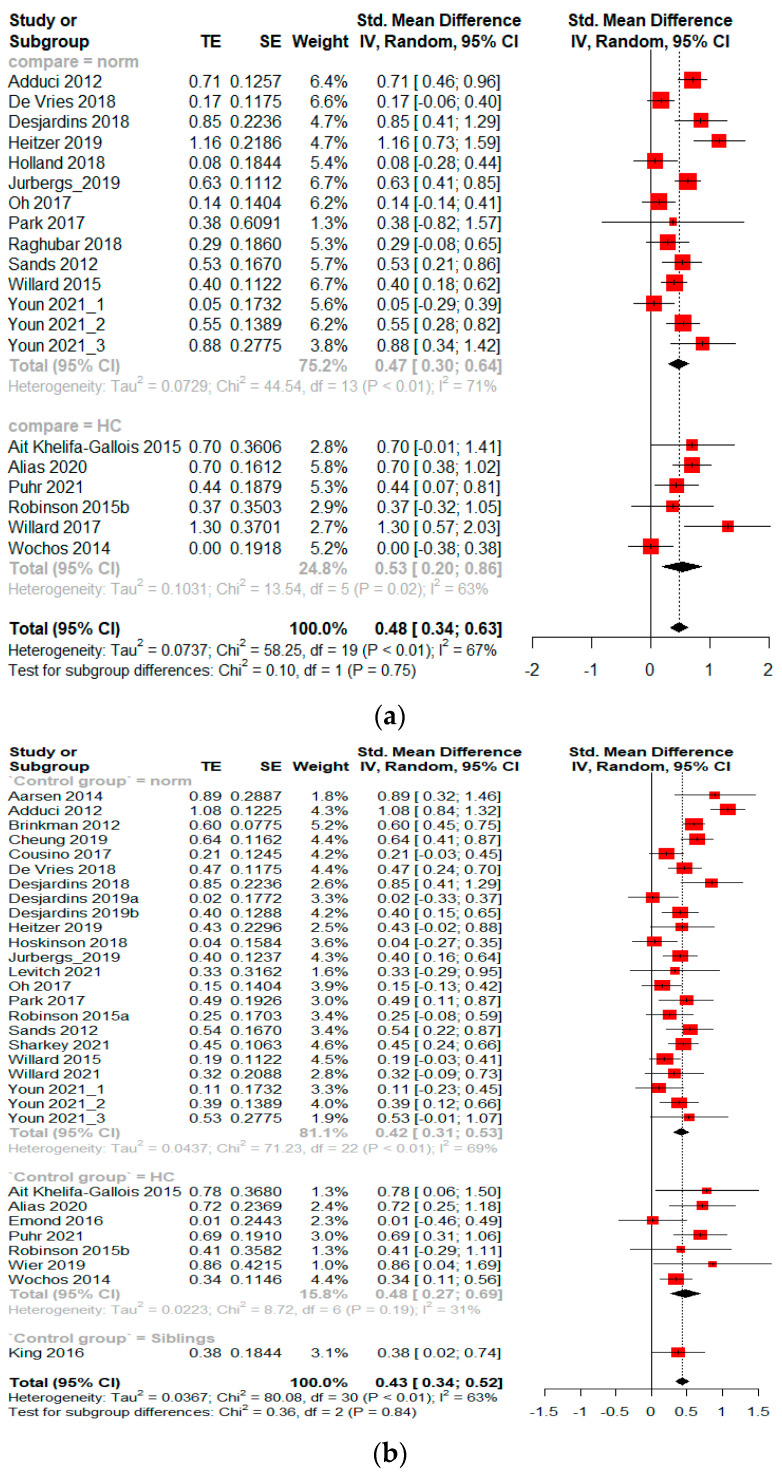

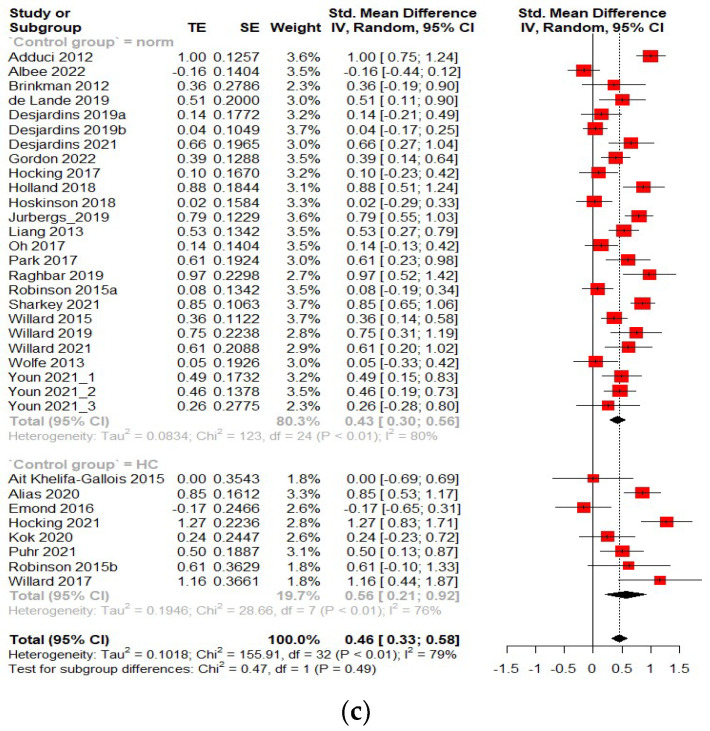
(**a**–**c**) Standard mean difference of pediatric brain tumor survivors having neurobehavioral impairment compared to the population norm and healthy controls. Youn et al. (2021) [83] reported more than one independent cohort in their study. HC: healthy control [35,36,37,38,39,40,41,42,43,44,45,46,47,48,50,51,52,53,54,55,56,57,58,61,62,65,66,67,68,69,70,71,72,75,76,77,78,80,81,82,83]. (**a**) Standard mean difference—attention problems; (**b**) Standard mean difference—emotional difficulties; (**c**) Standard mean difference—psychosocial problems.

The level of attention problems in PBTS was reported in 18 studies based on standard validated scales, with valid comparison groups. Among the studies, 12 compared PBTS to the population norm and six compared PBTS to healthy controls. There was no significant difference between the two comparison methods. The analysis revealed increased attention problems in PBTS compared to the population norm and healthy controls (Hedge’s g = 0.48 [95%CI 0.34–0.63], Figure 3a). There was a significant level of heterogeneity across the different studies (I^2^ = 67%, *p* < 0.01). The publication bias was not significant, as revealed by Egger’s test t (18) = 0.92, *p* = 0.369 (Figure S6a).

#### 3.3.2. Standard Mean Difference—Emotional Difficulties

The level of emotional difficulties in PBTS was reported in 29 studies based on standard validated scales, with valid comparison groups. Among the studies, 21 compared PBTS to the population norm, seven compared PBTS to healthy controls, and one study compared PBTS to their siblings. There were no significant differences between the comparison methods. The analysis revealed increased emotional difficulties in PBTS compared to the population norm and control groups (Hedge’s g = 0.43 [95%CI 0.34–0.52], Figure 3b). There was a significant level of heterogeneity across the different studies (I^2^ = 63%, *p* < 0.01). Notably, there was insignificant heterogeneity in the comparison with healthy controls (I^2^ = 31%), whereas the heterogeneity remained high in the subgroup that was compared with the population norm (I^2^ = 69%). No significant publication bias was observed, t (29) = −0.16, *p* = 0.877 (Figure S6b).

#### 3.3.3. Standard Mean Difference—Psychosocial Problems

The level of psychosocial problems in PBTS was reported in 32 studies based on standard validated scales. Among the studies, 25 compared PBTS to the population norm and seven studies compared PBTS to healthy controls. There was no significant difference between the two comparison methods. The analysis revealed an elevated level of psychosocial problems in PBTS compared to the population norm and control groups (Hedge’s g = 0.46 [95%CI 0.33–0.58], Figure 3c). There was a significant level of heterogeneity across the different studies (I^2^ = 79%, *p* < 0.01). No significant publication bias was identified t (31) = 0.35, *p* = 0.730 (Figure S6c).

### 3.4. Subgroup Analysis

#### 3.4.1. Reporting Method

The included studies were separated into subgroups according to the reporting method (self-report, parent-report, and teacher report). No significant differences were observed for attention problems and emotional difficulties regarding both their absolute risk and standard mean difference (Figure S1a–d). For psychosocial problems, the self-report subgroup showed lower absolute risk (3%) compared with the parent-report (13%) and teacher-report (40%) subgroups (χ^2^ = 9.58, *p* < 0.01), Figure S1e. There were no significant differences when comparing the standard mean differences of PBTS having psychosocial problems compared to population norms or healthy controls, among different reporting methods (Figure S1f). Significant high heterogeneity was observed in the parent-report subgroup across all measures. Low heterogeneity was only found in the self-report subgroup in the absolute risk/standard mean difference of attention problems, the standard mean difference of emotional difficulties, and absolute risk of psychosocial problems. However, heterogeneity remained high in other measures in subgroup analysis (Studies with neurobehavior measures based on more than one reporting method were separated into different categories as multiple subsamples. Thus, the pooled result in Figure S1a–f could be different from that in Figure 2 and Figure 3, as the same sample could be counted for multiple entries (e.g., self-report + parent-report) in this subgroup analysis).

#### 3.4.2. Treatment

Ten studies exclusively reported PBTS with a history of RT (RT-only) and three studies exclusively reported PBTS without a history of RT (no-RT). Thirty-seven studies reported a heterogenous sample including participants that both underwent RT and those that did not (mix-RT). Figures S2a–c and S3a–c demonstrated the subgroup analysis based on RT status, and there was no significant difference between the RT-only studies, no-RT studies, and mix-RT studies across different measures. To increase statistical power, we also pooled the standard mean difference of aspects of neurobehavioral impairment (social, emotional, attention) to examine the difference between RT-only and the no-RT group. However, there was no significant differences between those two groups regarding the standard mean difference of neurobehavioral impairment (Figure S4).

#### 3.4.3. Meta-Regression

A meta-regression was conducted with the standard mean difference and absolute risk as the criteria, and age at assessment, age at diagnosis, and follow-up time as the predictors, respectively. Three different aspects of neurobehavioral impairment (social, emotional, and attention) were pooled together to increase the standard mean difference. Age at assessment and age at diagnosis were not significant predictors of either absolute risk or standard mean difference of neurobehavioral impairment in PBTS (*p* > 0.3 in all regression models). A trend was identified whereby the follow-up time was associated with the standard mean difference (β = 0.17, *p* = 0.106), although it did not reach the significant level.

#### 3.4.4. Sensitivity Analysis

A sensitivity analysis was conducted through excluding studies with small sample size (*n* < 30) and/or were rated as having a ‘high’ risk of bias. The result revealed that there was no significant difference compared to the main analysis, see Figure S7a–f.

## 4. Discussion

As the survival of children with brain tumors has improved with advancements in cancer treatment, it becomes essential for healthcare professionals and childcare workers to have a better understanding of the long-term neurobehavioral sequelae of PBTS. This meta-analysis is one of the first to synthesize the recent evidence on the prevalence of neurobehavioral impairment in PBTS. The analysis showed that PBTS have a higher risk of neurobehavioral impairments compared to healthy subjects or the population norm. 18.9% and 15% of PBTS were found to have emotional difficulties and attention problems, respectively, when compared to a rate of 5.1% and 4.4% of the pediatric population with emotional problems and symptoms of inattention/hyperactivity according to a recent U.S. National Health Interview Survey [84].14.4% of PBTS were found to have psychosocial problems, compared to only 10.4% of children who were reported to have psychosocial problems according to a community sample of Dutch children [85].

Despite the well-reported detrimental effects of cranial radiotherapy on cognition and memory in PBTS, our study did not find significant differences in the rates of neurobehavioral impairments between children with or without cranial radiotherapy treatment, although these inconsistent findings might be related to the small sample sizes and high heterogeneity among studies. The impact of radiotherapy could vary due to irradiation dosage [40], tumor location/type [68,70] and follow-up time [77]. It is plausible that the neurobehavioral outcomes of PBTS are influenced primarily by the injury to the brain and the treatments received, as well as psychosocial and environmental factors. Having cancer in early childhood is an early unpleasant experience, as the presence of a life-threatening disease and the repeated invasive medical procedures can be very traumatic. These early childhood adversities might lead to neurobiological changes and increase the risk of emotional and behavioral impairments. Hence, it is essential to monitor the neurobehavioral functioning of PBTS regardless of whether they receive cranial radiotherapy or not.

For the long-term monitoring of neurobehavioral function in PBTS, the screening of attention problems and emotional difficulties could be achieved using self-reported and/or parent/proxy-reported questionnaires [86], as our study demonstrated that these questionnaires showed comparable rates of neurobehavioral impairment. More importantly, in older PBTS, self-report was found to be a valuable tool for psychosocial assessment, particularly in adolescents who might not want to discuss their symptoms in a clinical interview [87]. However, it is important to note that parent reporting is still an essential method for screening their children’s psychosocial problems, as we found that children tended to self-report lower rates of psychosocial problems. It is possible that PBTS with weak psychosocial skills might not be aware of their psychosocial needs, leading to under-reporting. To facilitate early identification of neurobehavioral impairment in long-term PBTS, survivorship programs should utilize both self-report and parent/proxy-report questionnaires for screening of those at risk of neurobehavioral problems. For specific subgroups of PBTS with low follow-up or low attendance at survivorship clinics, such as adolescents [88] or those from underprivileged families, clinicians should consider distributing questionnaires electronically. Although self-/parent-reports cannot be substitutes for objective neuropsychological assessments, they can certainly be used as a screening tool to enhance clinical care and better identify those in need of psychological and psychiatric services and support.

**Figure 4:**
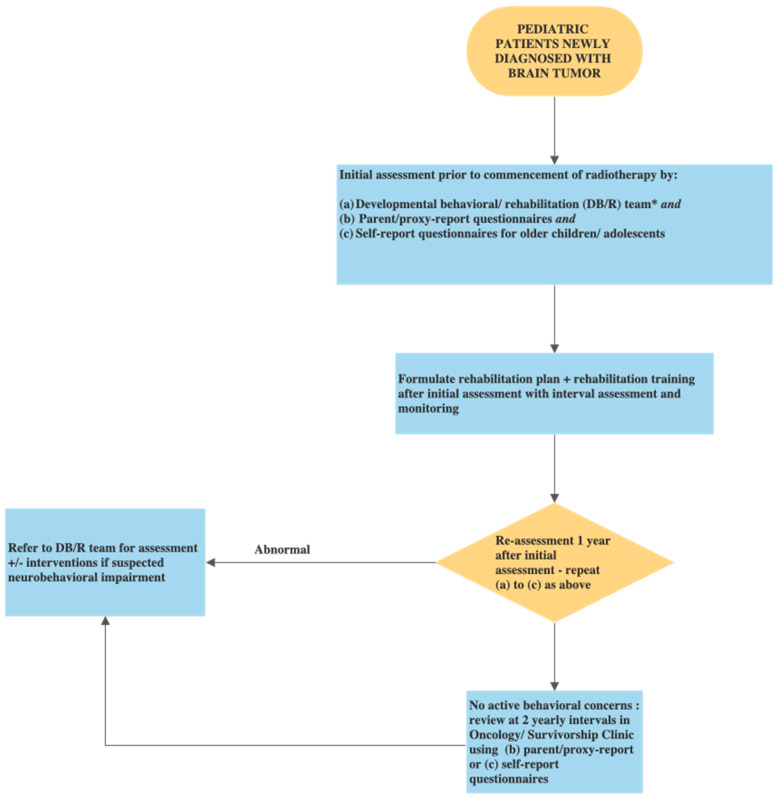
Flow chart: Monitoring for neurobehavioral impairment in children with brain tumor. * Developmental behavioral/rehabilitation (DB/R) team includes: developmental behavioral pediatrician or equivalent, clinical psychologist (preferably neuropsychologist), occupational therapist, physiotherapist, speech therapist and medical social worker. The DB/R team is supported by the child psychiatrist (on consultation basis) and works in close collaboration with the community pediatricians and educators e.g., school social workers/educational psychologists.

In order to monitor the trajectory of neurobehavioral problems among PBTS, we propose that all children newly diagnosed with brain tumors should have comprehensive neurocognitive and behavioral evaluation by healthcare professionals. The initial assessment should include diagnostic interviews conducted by healthcare professionals as well as using parent and self-report questionnaires. All PBTS should have regular monitoring for neurobehavioral impairment using parent and self-report questionnaires (Figure 4). For parent/proxy-report questionnaires, the Child Behavioral Checklist (CBCL) was most frequently used among the studies included in this meta-analysis. Other parent/proxy-report questionnaires included the Adaptive Behavior Assessment System-Second Edition (ABAS-II), the Behavior Ratings of Executive Function (BRIEF) or the Behavior Assessment System for Children (BASC). Self-report questionnaires such as the Youth self-report (YSR) can be used for children aged 11 to 18 years (File S2). Children with abnormal scores should be referred for detailed assessment and referral for psychiatric evaluation and interventions. Timely interventions such as psychotherapy or problem-solving therapy were found to be beneficial for PBTS with emotional difficulties or psychosocial problems [89,90]. Social skills training was found to improve social competence in PBTS [91]. For childhood cancer survivors with attention problems, psychostimulants such as methylphenidate was found to significantly improve their sustained attention [92].

This study had several limitations that need to be considered. There was significant heterogeneity among the included studies due to variations in patient characteristics and types of treatments across studies. High heterogeneity was also reported by Schulte et al., 2019 [21] in a systematic review that examined social attainment outcomes in survivors of pediatric CNS tumors from 2011 to 2018. Some of the heterogeneity could be due to the comparison group and reporting method. The healthy control subgroup and self-report subgroup in our analysis appeared to show lower heterogeneity in some measures. However, that could be due to the small sample size in these subgroups (df < 10). Possible other sources of heterogeneity include the type and severity of the brain tumor, the assessment tools, and different treatments. Due to the high heterogeneity, the results from comparing subgroups shall be interpreted with caution, as the grouping factors (e.g., RT status) could be confounded by other variables. Although the asymmetry tests for funnel plots did not reach the significant level in our analysis, publication bias is another inherent limitation in this meta-analysis, as PBTS with neurobehavioral problems have a higher likelihood of being reported than studies with negative findings. Our meta-analysis included parent- and self-reported data and clinical diagnoses of neurobehavioral impairment in PBTS using different screening or diagnostic tools. However, we did not include studies using task-based assessment of neurobehavioral outcome, as the majority of these assessments were conducted for research purposes rather than in clinical practice. Therefore, our recruitment strategy and inclusion criteria might be a potential source of selection bias. Longitudinal studies with a larger sample size of PBTS using diagnostic interviews and detailed behavioral assessments need to be conducted to validate our study findings.

## 5. Conclusions

In conclusion, neurobehavioral impairments, including emotional, psychosocial, and attention problems, are more common in PBTS. Survivor-reported or proxy-reported questionnaires might serve as good screening tools for PBTS at risk of neurobehavioral problems. Survivorship programs should offer long-term monitoring of neurobehavioral function in PBTS. Future studies should focus on exploring potential interventions for PBTS at risk of neurobehavioral impairment. 

## Figures and Tables

**Table 1:** Characteristics of the included studies.

Study	Tumor Type	Assessment Tool	Age at Diagnosis	Age at Assessment	Sample Size	Psychosocial Measure	Emotion Measure	Attention Measure	Report Type	Comparison Group	Risk of Bias	Radio Therapy	Region
Aarsen 2014 [35]	low grade tectal tumor	CBCL & YSR	10.02	14.30	12	N	Y	N	parent-& self-reports	norm	Low	N	EU
Adduci 2012 [36]	mix	CBCL & VABS	6.76	9.47	64	Y	Y	Y	parent-report	norm	Low	mix	EU
Ait Khelifa-Gallois 2015 [37]	pilocytic astrocytoma	scale adapted from CBCL, SDQ and Conners Scale	6.80	15.10	18	Y	Y	Y	parent-report	HC	Low	N	EU
Albee 2022 [38]	mix	SSIS	8.87	10.62	51	Y	N	N	parent-report	norm	Low	mix	NA
Alias 2020 [39]	mix	CBCL	7.20	12.50	38	Y	Y	Y	parent-report	HC	Low	mix	Asia
Brinkman 2012 [40]	embryonal tumor	CBCL	10.70	11.00	169	Y	Y	N	parent-report	norm	Low	Y	NA
Cheung 2019 [41]	mix	CES-DC	9.70	11.70	77	N	Y	N	self-report	norm	Medium	mix	Asia
Cousino 2017 [42]	mix	BASC	6.16	14.30	65	N	Y	N	parent- report	norm	Low	mix	NA
De Lande 2019 [43]	low-grade glioma	VABS	7.16	12.08	56	Y	N	N	parent- report	norm	Low	mix	EU
De Vries 2018 [44]	mix	BRIEF	6.90	13.80	73	N	Y	Y	parent-report	norm	Low	mix	EU
Desjardins 2018 [45]	mix	CBCL	9.79	10.79	20	N	Y	Y	parent-report	norm	Medium	mix	NA
Desjardins 2019a [46]	mix	SSRS & BRIEF	5.87	10.41	32	Y	Y	N	parent-report	norm	Low	mix	NA
Desjardins 2019b [47]	mix	SSRS & BRIEF	5.22	11.21	91	Y	Y	N	parent-report	norm	Low	mix	NA
Desjardins 2021 [48]	mix	CBCL	6.09	14.00	26	Y	N	N	parent-report	norm	Low	mix	NA
Dessens 2016 [49]	mix	CBCL	5.80	11.70	13	Y	Y	Y	child- & parent-reports	norm	Low	mix	EU
Emond 2016 [50]	mix	SSIS, SRS & SDQ	6.71	12.59	33	Y	Y	N	self-, parent- & teacher-reports	HC	Medium	mix	EU
Gordon 2022 [51]	mix	SPPC & NTEM	4.14	10.59	65	Y	N	N	self- & parent-reports	norm	Low	mix	NA
Hardy 2018 [24]	mix	ADHD-RS-IV	6.20	12.00	105	N	N	Y	parent-& teacher-reports	norm	Low	mix	NA
Heitzer 2019 [52]	low-grade glioma	CBCL	0.51	9.90	19	N	Y	Y	parent-report	norm	Low	mix	NA
Hocking 2017 [53]	mix	SSIS	5.66	14.46	36	Y	N	N	parent-report	norm	Low	mix	NA
Hocking 2021 [54]	mix	SRS	6.10	13.72	54	Y	N	N	parent-report	HC	Low	mix	NA
Holland 2018 [55]	medulloblastoma	CBCL	8.02	13.96	33	Y	N	Y	parent-report	norm	Low	Y	NA
Hoskinson 2018 [56]	mix	ABAS-II; BASC	10.72	12.76	40	Y	Y	N	parent-report	norm	Low	mix	NA
Jurbergs 2019 [57]	mix	ABAS-II CBCL	2.39	4.52	67	Y	Y	Y	parent-report	norm	High	mix	NA
King 2016 [58]	medulloblastoma	BSI	9.00	NR	198	N	Y	N	self-report	Siblings	Low	mix	NA
Kok 2020 [59]	mix	CBCL	9.17	8.33	21	Y	N	N	parent-& teacher-reports	HC	Medium	N	EU
Kristiansen 2019 [60]	low-grade astrocytoma	BYI, BDI, BAI	8.70	20.8	7	Y	Y	N	self-report	norm	Medium	mix	EU
Levitch 2021 [61]	mix	BASC	2.98	10.23	10	N	Y	N	parent-report	norm	Low	mix	NA
Liang 2013 [62]	intracranial germ cell tumor	ABAS	11.90	17.70	56	Y	N	N	parent-report	norm	Low	Y	Asia
Moitra & Armstrong 2013 [63]	mix	SCARED–C	6.56	11.40	91	N	Y	N	self-report	norm	Medium	N/A	NA
Nelson 2021 [64]	posterior fossa brain tumor	CBCL	11.32	5.00	28	Y	Y	N	parent-report	norm	Low	mix	NA
Oh 2017 [65]	mix	K-PRC	10.06	10.33	51	Y	Y	Y	parent-report	norm	High	Y	Asia
Park 2017 [66]	intracranial germ cell tumor	CBCL	12.30	12.60	27	Y	Y	Y	parent-report	norm	Medium	Y	Asia
Puhr 2021 [67]	mix	CBCL & YSR	6.80	15.70	48	Y	Y	Y	parent- & self-report	HC	Low	mix	EU
Raghubar 2018 [68]	mix	BASC	9.12	11.54	29	N	N	Y	parent-report	norm	Low	mix	NA
Raghbar 2019 [69]	mix	ABAS-II	6.39	13.37	114	Y	N	N	parent-report	norm	Low	Y	NA
Robinson 2015a [70]	mix	ABAS-II	10.67	10.72	47	Y	Y	N	parent-report	norm	Low	Y	NA
Robinson 2015b [71]	mix	YSR &CBCL	6.94	12.60	17	Y	Y	Y	self- & parent-reports	HC	Low	mix	NA
Sands 2012 [72]	mix	NFI	8.80	23.60	35	N	Y	Y	parent-report & Self-report	norm	Low	Y	NA
Schulte 2018 [73]	mix	CBCL	N/A	15.00	665	Y	N	N	parent-report	norm *	Low	mix	NA
Shabason 2019 [74]	mix	ADHD diagnosis	8.15	15.50	528	N	N	Y	clinical diagnosis	norm	Low	mix	NA
Sharkey 2021 [75]	mix	CBCL	6.57	12.60	89	Y	Y	N	parent-report	norm	Low	mix	NA
Wier 2019 [76]	mix	CBCL	N/A	12.83	11	Y	N	N	parent-report	HC	Low	mix	NA
Willard 2015 [77]	low-grade glioma	CBCL	6.80	8.90	80	Y	Y	Y	parent-report	norm	Low	Y	NA
Willard 2017 [78]	mix	CBCL	5.19	11.79	10	Y	N	Y	parent-report	HC	Low	mix	NA
Willard 2019 [79]	mix	SEARS	8.53	14.70	20	Y	N	N	self-, parent-& teacher-reports	norm	Low	mix	NA
Willard 2021 [80]	mix	NTEM &BASC	3.61	5.46	23	Y	Y	N	parent-report	norm	Low	mix	NA
Wochos 2014 [81]	mix	BRIEF	4.68	5.81	62	N	Y	Y	parent-report	HC	Low	mix	NA
Wolfe 2013 [82]	mix	SSIS & BRIEF	4.50	9.10	24	Y	N	N	parent-& self-reports	norm	Low	mix	NA
Youn 2021 [83]	mix	CBCL	9.30	0.60	33	Y	Y	Y	parent-report	norm	Low	Y	Asia

CBCL: Child Behavior Checklist; YSR: Youth Self-Report; VABS: Vineland Adaptive Behavior Scales; SDQ: Strengths and Difficulties Questionnaire; SSIS: Psychosocial Skills Improvement System; CES-DC: Center for Epidemiological Studies Depression Scale for Children; BASC: Behavior Assessment System for Children; BRIEF: Behavior Ratings of Executive Function; SSRS: Psychosocial Skills Rating System; SPPC: Self-Perception Profile for Children; NTEM: NIH Toolbox—Emotion Measures; ADHD-RS-IV: ADHD Rating Scale-IV; SRS: The Psychosocial Responsiveness Scale; ABAS-II: Adaptive Behavior Assessment System-Second Edition; BSI: Brief Symptom Inventory–18; BAI: Beck Anxiety Inventory; BDI: Beck Depression Inventory; SCARED–C: Screen for Child Anxiety Related Emotional Disorders–Child version; BYI: Beck Youth Inventories; K-PRC: Korean Personality Rating scale for Children; NFI: Neuropsychological Functioning Inventory; SEARS: Psychosocial-Emotional Assets and Resilience Scales; CPRS: Psychosocial-Emotional Assets and Resilience Scales; Y: yes; N:no; HC: healthy control; NA: North America; EU: Europe. N/A: not available. * Solid tumor was used as the comparison group in the study to better synthesize the result, whereas population norm was used in the meta-analysis.

## Data Availability

The raw data and statistical code for conducting this meta-analysis is available upon request.

## Supplementary Materials

The following supporting information can be downloaded at: https://www.mdpi.com/article/10.3390/cancers14133269/s1, File S1: Searching String for Each Database; File S2: Assessment Tools Used in the Reviewed Studies; Figure S1: The absolute risk and standard mean difference (as compared to population norm or healthy controls) of pediatric brain tumor survivors having neurobehavioral impairment according to different reporting methods; Figure S2: Absolute risk of neurobehavioral impairment in paediatric brain tumor survi-vors by different radio therapy status. Figure S3: Standard mean differences of neurobehavioral impairment in paediatric brain tumor survivors compared to healthy controls and population norm by different radio therapy status; Figure S4: The pooled standard mean difference of pediatric brain tumor survivors having neu-robehavioral impairment in patients with or without radiotherapy; Figure S5: Funnel plot of logit transformation of absolute risk of neurobehavioral im-pairment in paediatric brain tumor survivors; Figure S6: Funnel plot of standard mean differences of neurobehavioral impairment in paediatric brain tumor survivors compared to population norm or healthy control; Figure S7: The absolute risk and standard mean difference (as compared to population norm or healthy controls) of pediatric brain tumor survivors having neurobehavioral impairment ac-cording to different reporting methods, excluding studies with high risk of bias and low sample size (n < 30). References [91,92,93,94,95,96,97,98,99,100,101,102,103,104,105,106,107,108,109,110,111,112,113,114] are cited in Supplementary Materials.

## References

[1] Lamba N., Groves A., Torre M., Yeo K.K., Iorgulescu J.B. (2022). The epidemiology of primary and metastatic brain tumors in infancy through childhood. J. Neurooncol..

[2] Pui C.-H., Gajjar A.J., Kane J.R., Qaddoumi I.A., Pappo A.S. (2011). Challenging issues in pediatric oncology. Nat. Rev. Clin. Oncol..

[3] Perreault S., Desjardins L., Scheinemann K. (2022). Long Term Sequelae.

[4] Palmer S.L., Goloubeva O., Reddick W.E., Glass J.O., Gajjar A., Kun L., Merchant T.E., Mulhern R.K. (2001). Patterns of intellectual development among survivors of pediatric medulloblastoma: A longitudinal analysis. J. Clin. Oncol..

[5] Spiegler B.J., Kennedy K., Maze R., Greenberg M.L., Weitzman S., Hitzler J.K., Nathan P.C. (2006). Comparison of long-term neurocognitive outcomes in young children with acute lymphoblastic leukemia treated with cranial radiation or high-dose or very high-dose intravenous methotrexate. J. Clin. Oncol..

[6] Ellenberg L., Liu Q., Gioia G., Yasui Y., Packer R.J., Mertens A., Donaldson S.S., Stovall M., Kadan-Lottick N., Armstrong G. (2009). Neurocognitive Status in Long-Term Survivors of Childhood CNS Malignancies: A Report from the Childhood Cancer Survivor Study. Neuropsychology.

[7] Brinkman T.M., Krasin M.J., Liu W., Armstrong G.T., Ojha R.P., Sadighi Z.S., Gupta P., Kimberg C., Srivastava D., Merchant T.E. (2016). Long-term neurocognitive functioning and social attainment in adult survivors of pediatric CNS tumors: Results from the St Jude Lifetime Cohort Study. J. Clin. Oncol..

[8] Tso W.W.Y., Hui E.S.K., Lee T.M.C., Liu A.P.Y., Ip P., Vardhanabhuti V., Cheng K.K.F., Fong D.Y.T., Chang D.H.F., Ho F.K.W. (2021). Brain Microstructural Changes Associated With Neurocognitive Outcome in Intracranial Germ Cell Tumor Survivors. Front. Oncol..

[9] Tso W.W.Y., Liu A.P.Y., Lee T.M.C., Cheuk K.L., Shing M.K., Luk C.W., Ling S.C., Ku D.T.L., Li K., Yung A.W.Y. (2019). Neurocognitive function, performance status, and quality of life in pediatric intracranial germ cell tumor survivors. J. Neurooncol..

[10] Zureick A.H., Evans C.L., Niemierko A., Grieco J.A., Nichols A.J., Fullerton B.C., Hess C.B., Goebel C.P., Gallotto S.L., Weyman E.A. (2018). Left hippocampal dosimetry correlates with visual and verbal memory outcomes in survivors of pediatric brain tumors. Cancer.

[11] Morrall M., Reed-Berendt R., Moss K., Stocks H., Houston A.L., Siddell P., Picton S., Grundy R. (2019). Neurocognitive, academic and functional outcomes in survivors of infant ependymoma (UKCCSG CNS 9204). Childs Nerv. Syst..

[12] Lassaletta A., Bouffet E., Mabbott D., Kulkarni A.V. (2015). Functional and neuropsychological late outcomes in posterior fossa tumors in children. Childs Nerv. Syst..

[13] Levitch C.F., Holland A.A., Bledsoe J., Kim S.Y., Barnett M., Ramjan S., Sands S.A. (2022). Comparison of neuropsychological functioning in pediatric posterior fossa tumor survivors: Medulloblastoma, low-grade astrocytoma, and healthy controls. Pediatr Blood Cancer.

[14] Kahalley L.S., Peterson R., Ris M.D., Janzen L., Okcu M.F., Grosshans D.R., Ramaswamy V., Paulino A.C., Hodgson D., Mahajan A. (2020). Superior Intellectual Outcomes After Proton Radiotherapy Compared With Photon Radiotherapy for Pediatric Medulloblastoma. J. Clin. Oncol..

[15] Centers for Disease Control and Prevention Data and Statistics on Children’s Mental Health. https://www.cdc.gov/childrensmentalhealth/data.html.

[16] Asarnow R.F., Newman N., Weiss R.E., Su E. (2021). Association of Attention-Deficit/Hyperactivity Disorder Diagnoses with Pediatric Traumatic Brain Injury: A Meta-analysis. JAMA Pediatr..

[17] Noll R.B., Gartstein M.A., Vannatta K., Correll J., Bukowski W.M., Davies W.H. (1999). Social, emotional, and behavioral functioning of children with cancer. Pediatrics.

[18] Bonanno M., Bourque C.J., Aramideh J., Cloutier N., Dumont E., Gomez-Tyo M., Julien-Lacoste A., Kosir U., Provost C., Laverdiere C. (2021). Articulating viewpoints to better define and respond to the needs of adolescents and young adult survivors of pediatric brain tumors. J. Psychosoc. Oncol..

[19] Hocking M.C., McCurdy M., Turner E., Kazak A.E., Noll R.B., Phillips P., Barakat L.P. (2015). Social competence in pediatric brain tumor survivors: Application of a model from social neuroscience and developmental psychology. Pediatr Blood Cancer.

[20] Salley C.G., Hewitt L.L., Patenaude A.F., Vasey M.W., Yeates K.O., Gerhardt C.A., Vannatta K. (2015). Temperament and social behavior in pediatric brain tumor survivors and comparison peers. J. Pediatr. Psychol..

[21] Schulte F., Kunin-Batson A.S., Olson-Bullis B.A., Banerjee P., Hocking M.C., Janzen L., Kahalley L.S., Wroot H., Forbes C., Krull K.R. (2019). Social attainment in survivors of pediatric central nervous system tumors: A systematic review and meta-analysis from the Children’s Oncology Group. J. Cancer Surviv..

[22] Pastore V., Colombo K., Villa F., Galbiati S., Adduci A., Poggi G., Massimino M., Recla M., Liscio M., Strazzer S. (2013). Psychological and adjustment problems due to acquired brain lesions in pre-school-aged patients. Brain Inj..

[23] Poggi G., Liscio M., Galbiati S., Adduci A., Massimino M., Gandola L., Spreafico F., Clerici C.A., Fossati-Bellani F., Sommovigo M. (2005). Brain tumors in children and adolescents: Cognitive and psychological disorders at different ages. Psychooncology.

[24] Hardy K.K., Willard V.W., Gioia A., Sharkey C., Walsh K.S. (2018). Attention-mediated neurocognitive profiles in survivors of pediatric brain tumors: Comparison to children with neurodevelopmental ADHD. Neuro-Oncology.

[25] Gragert M.N., Ris M.D. (2011). Neuropsychological late effects and rehabilitation following pediatric brain tumor. J. Pediatr. Rehabil. Med..

[26] Vannatta K., Gartstein M.A., Short A., Noll R.B. (1998). A controlled study of peer relationships of children surviving brain tumors: Teacher, peer, and self ratings. J. Pediatr. Psychol..

[27] Vannatta K., Gerhardt C.A., Wells R.J., Noll R.B. (2007). Intensity of CNS treatment for pediatric cancer: Prediction of social outcomes in survivors. Pediatr Blood Cancer.

[28] Trendowski M.R., Baedke J.L., Sapkota Y., Travis L.B., Zhang X., El Charif O., Wheeler H.E., Leisenring W.M., Robison L.L., Hudson M.M. (2021). Clinical and genetic risk factors for radiation-associated ototoxicity: A report from the Childhood Cancer Survivor Study and the St. Jude Lifetime Cohort. Cancer.

[29] Rosenthal R., Cooper H., Hedges L. (1994). Parametric measures of effect size. Handb. Res. Synth..

[30] Higgins J.P.T., Altman D.G., Gøtzsche P.C., Jüni P., Moher D., Oxman A.D., Savović J., Schulz K.F., Weeks L., Sterne J.A.C. (2011). The Cochrane Collaboration’s tool for assessing risk of bias in randomised trials. BMJ.

[31] Peters J.L., Sutton A.J., Jones D.R., Abrams K.R., Rushton L. (2006). Comparison of two methods to detect publication bias in meta-analysis. JAMA.

[32] Egger M., Smith G.D., Schneider M., Minder C. (1997). Bias in meta-analysis detected by a simple, graphical test. BMJ.

[33] Balduzzi S., Rücker G., Schwarzer G. (2019). How to perform a meta-analysis with R: A practical tutorial. Evid.-Based Ment. Health.

[34] Selçuk A.A. (2019). A guide for systematic reviews: PRISMA. Turk. Arch. Otorhinolaryngol..

[35] Aarsen F.K., Arts W.F., Van Veelen-Vincent M.L., Lequin M.H., Catsman-Berrevoets C.E. (2014). Long-term outcome in children with low grade tectal tumours and obstructive hydrocephalus. Eur. J. Paediatr. Neurol..

[36] Adduci A., Jankovic M., Strazzer S., Massimino M., Clerici C., Poggi G. (2012). Parent-child communication and psychological adjustment in children with a brain tumor. Pediatric Blood Cancer.

[37] Ait Khelifa-Gallois N., Laroussinie F., Puget S., Sainte-Rose C., Dellatolas G. (2015). Long-term functional outcome of patients with cerebellar pilocytic astrocytoma surgically treated in childhood. Brain Inj..

[38] Albee M., Allende S., Cosgrove V., Hocking M.C. (2022). A prospective study of social competence in survivors of pediatric brain and solid tumors. Pediatric Blood Cancer.

[39] Alias H., Morthy S.K., Zakaria S.Z.S., Muda Z., Tamil A.M. (2020). Behavioral outcome among survivors of childhood brain tumor: A case control study. BMC Pediatrics.

[40] Brinkman T.M., Palmer S.L., Chen S., Zhang H., Evankovich K., Swain M.A., Bonner M.J., Janzen L., Knight S., Armstrong C.L. (2012). Parent-reported social outcomes after treatment for pediatric embryonal tumors: A prospective longitudinal study. J. Clin. Oncol..

[41] Cheung A.T., Li W.H.C., Ho L.L.K., Ho K.Y., Chiu S., Chan C.-F.G., Chung O.K. (2019). Impact of brain tumor and its treatment on the physical and psychological well-being, and quality of life amongst pediatric brain tumor survivors. Eur. J. Oncol. Nurs..

[42] Cousino M.K., Hazen R., Josie K.L., Laschinger K., de Blank P., Taylor H.G. (2017). Childhood cancer and brain tumor late effects: Relationships with family burden and survivor psychological outcomes. J. Clin. Psychol. Med. Settings.

[43] De Lande R.V., Maurice-Stam H., Marchal J., Vuurden D.V., Vandertop W., Grootenhuis M., Schouten-van Meeteren A. (2019). Adaptive behavior impaired in children with low-grade glioma. Pediatric Blood Cancer.

[44] De Vries M., De Ruiter M., Oostrom K., Schouten-Van Meeteren A., Maurice-Stam H., Oosterlaan J., Grootenhuis M. (2018). The association between the behavior rating inventory of executive functioning and cognitive testing in children diagnosed with a brain tumor. Child Neuropsychol..

[45] Desjardins L., Thigpen J.C., Kobritz M., Bettis A.H., Gruhn M.A., Ichinose M., Hoskinson K., Fraley C., Vreeland A., McNally C. (2018). Parent reports of children’s working memory, coping, and emotional/behavioral adjustment in pediatric brain tumor patients: A pilot study. Child Neuropsychol..

[46] Desjardins L., Barrera M., Chung J., Cataudella D., Janzen L., Bartels U., Downie A., Fairclough D. (2019). Are we friends? Best friend nominations in pediatric brain tumor survivors and associated factors. Supportive Care Cancer.

[47] Desjardins L., Barrera M., Schulte F., Chung J., Cataudella D., Janzen L., Bartels U., Downie A. (2019). Predicting social withdrawal, anxiety and depression symptoms in pediatric brain tumor survivors. J. Psychosoc. Oncol..

[48] Desjardins L., Lai M.-C., Vorstman J., Bartels U., Barrera M. (2021). A Novel Approach to Understanding Social Behaviors in Pediatric Brain Tumor Survivors: A Pilot Study. J. Pediatric Psychol..

[49] Dessens A.B., van Herwerden M.C., Aarsen F.K., Birnie E., Catsman-Berrevoets C.E. (2016). Health-related quality of life and emotional problems in children surviving brain tumor treatment: A descriptive study of 2 cohorts. Pediatric Hematol. Oncol..

[50] Emond A., Edwards L., Peacock S., Norman C., Evangeli M. (2016). Social competence in children and young people treated for a brain tumour. Supportive Care Cancer.

[51] Gordon M.L., Means B., Jurbergs N., Conklin H.M., Gajjar A., Willard V.W. (2022). Social Problem Solving in Survivors of Pediatric Brain Tumor. J. Pediatric Psychol..

[52] Heitzer A.M., Ashford J.M., Hastings C., Liu A.P., Wu S., Bass J.K., Vestal R., Hoehn M., Chiang J., Ghazwani Y. (2019). Neuropsychological outcomes of patients with low-grade glioma diagnosed during the first year of life. J. Neuro-Oncol..

[53] Hocking M.C., Quast L.F., Brodsky C., Deatrick J.A. (2017). Caregiver perspectives on the social competence of pediatric brain tumor survivors. Supportive Care Cancer.

[54] Hocking M.C., Albee M., Brodsky C., Shabason E., Wang L., Schultz R.T., Herrington J. (2021). Face processing and social functioning in pediatric brain tumor survivors. J. Pediatric Psychol..

[55] Holland A.A., Colaluca B., Bailey L., Stavinoha P.L. (2018). Impact of attention on social functioning in pediatric medulloblastoma survivors. Pediatric Hematol. Oncol..

[56] Hoskinson K.R., Wolfe K.R., Yeates K.O., Mahone E.M., Cecil K.M., Ris M.D. (2018). Predicting changes in adaptive functioning and behavioral adjustment following treatment for a pediatric brain tumor: A report from the Brain Radiation Investigative Study Consortium. Psycho-Oncology.

[57] Jurbergs N., Harman J.L., Kenney A.E., Semenkovich K., Molnar A.E., Willard V.W. (2019). Cognitive and psychosocial development in young children with brain tumors: Observations from a clinical sample. Children.

[58] King A.A., Seidel K., Di C., Leisenring W.M., Perkins S.M., Krull K.R., Sklar C.A., Green D.M., Armstrong G.T., Zeltzer L.K. (2017). Long-term neurologic health and psychosocial function of adult survivors of childhood medulloblastoma/PNET: A report from the Childhood Cancer Survivor Study. Neuro-Oncology.

[59] Kok T.B., Koerts J., Lemiere J., Post W.J., de Bont E.S., Gidding C., Happé F., Jacobs S., Oostrom K., Schieving J. (2020). Social competence in newly diagnosed pediatric brain tumor patients. Pediatric Hematol. Oncol..

[60] Kristiansen I., Strinnholm M., Strömberg B., Frisk P. (2019). Clinical characteristics, long-term complications and health-related quality of life (HRQoL) in children and young adults treated for low-grade astrocytoma in the posterior fossa in childhood. J. Neuro-Oncol..

[61] Levitch C.F., Malkin B., Latella L., Guerry W., Gardner S.L., Finlay J.L., Sands S.A. (2021). Long-term neuropsychological outcomes of survivors of young childhood brain tumors treated on the Head Start II protocol. Neuro-Oncol. Pract..

[62] Liang S.-Y., Yang T.-F., Chen Y.-W., Liang M.-L., Chen H.-H., Chang K.-P., Shan I.-K., Chen Y.-S., Wong T.-T. (2013). Neuropsychological functions and quality of life in survived patients with intracranial germ cell tumors after treatment. Neuro-Oncology.

[63] Moitra E., Armstrong C.L. (2013). Neural substrates for heightened anxiety in children with brain tumors. Dev. Neuropsychol..

[64] Baron Nelson M.C., O’Neil S.H., Tanedo J., Dhanani S., Malvar J., Nuñez C., Nelson M.D., Tamrazi B., Finlay J.L., Rajagopalan V. (2021). Brain biomarkers and neuropsychological outcomes of pediatric posterior fossa brain tumor survivors treated with surgical resection with or without adjuvant chemotherapy. Pediatric Blood Cancer.

[65] Oh Y., Seo H., Sung K.W., Joung Y.S. (2017). The effects of attention problems on psychosocial functioning in childhood brain tumor survivors: A 2-year postcraniospinal irradiation follow-up. J. Pediatric Hematol. Oncol..

[66] Park Y., Yu E.-S., Ha B., Park H.-J., Kim J.-H., Kim J.-Y. (2017). Neurocognitive and psychological functioning of children with an intracranial germ cell tumor. Cancer Res. Treat. Off. J. Korean Cancer Assoc..

[67] Puhr A., Ruud E., Anderson V., Due-Tønnessen B.J., Skarbø A.-B., Finset A., Andersson S. (2021). Executive Function and Psychosocial Adjustment in Adolescent Survivors of Pediatric Brain Tumor. Dev. Neuropsychol..

[68] Raghubar K.P., Mahone E.M., Yeates K.O., Ris M.D. (2018). Performance-based and parent ratings of attention in children treated for a brain tumor: The significance of radiation therapy and tumor location on outcome. Child Neuropsychol..

[69] Raghubar K.P., Orobio J., Ris M.D., Heitzer A.M., Roth A., Brown A.L., Okcu M.F., Chintagumpala M., Grosshans D.R., Paulino A.C. (2019). Adaptive functioning in pediatric brain tumor survivors: An examination of ethnicity and socioeconomic status. Pediatric Blood Cancer.

[70] Robinson K.E., Wolfe K.R., Yeates K.O., Mahone E.M., Cecil K.M., Ris M.D. (2015). Predictors of adaptive functioning and psychosocial adjustment in children with pediatric brain tumor: A report from the brain radiation investigative study consortium. Pediatric Blood Cancer.

[71] Robinson K.E., Pearson M.M., Cannistraci C.J., Anderson A.W., Kuttesch J.F., Wymer K., Smith S.E., Park S., Compas B.E. (2015). Functional neuroimaging of working memory in survivors of childhood brain tumors and healthy children: Associations with coping and psychosocial outcomes. Child Neuropsychol..

[72] Sands S.A., Zhou T., O’Neil S.H., Patel S.K., Allen J., Cullen P.M., Kaleita T.A., Noll R., Sklar C., Finlay J.L. (2012). Long-term follow-up of children treated for high-grade gliomas: Children’s oncology group L991 final study report. J. Clin. Oncol..

[73] Schulte F., Brinkman T.M., Li C., Fay-McClymont T., Srivastava D.K., Ness K.K., Howell R.M., Mueller S., Wells E., Strother D. (2018). Social adjustment in adolescent survivors of pediatric central nervous system tumors: A report from the C hildhood C ancer S urvivor S tudy. Cancer.

[74] Shabason E.K., Brodsky C., Baran J., Isaac L., Minturn J.E., Ginsberg J.P., Hobbie W., Fisher M., Blum N., Hocking M.C. (2019). Clinical diagnosis of attention-deficit/hyperactivity disorder in survivors of pediatric brain tumors. J. Neuro-Oncol..

[75] Sharkey C.M., Mullins L.L., Clawson A.H., Gioia A., Hawkins M.A., Chaney J.M., Walsh K.S., Hardy K.K. (2021). Assessing neuropsychological phenotypes of pediatric brain tumor survivors. Psycho-Oncology.

[76] Wier R., Aleksonis H.A., Pearson M.M., Cannistraci C.J., Anderson A.W., Kuttesch J.F., Compas B.E., Hoskinson K.R. (2019). Fronto-limbic white matter microstructure, behavior, and emotion regulation in survivors of pediatric brain tumor. J. Neuro-Oncol..

[77] Willard V.W., Conklin H.M., Wu S., Merchant T.E. (2015). Prospective longitudinal evaluation of emotional and behavioral functioning in pediatric patients with low-grade glioma treated with conformal radiation therapy. J. Neuro-Oncol..

[78] Willard V.W., Allen T.M., Hardy K.K., Bonner M.J. (2017). Social functioning in survivors of pediatric brain tumors: Contribution of neurocognitive and social-cognitive skills. Child. Health Care.

[79] Willard V.W., Russell K.M., Long A., Phipps S. (2019). The impact of connectedness on social functioning in youth with brain tumors. Pediatric Blood Cancer.

[80] Willard V.W., Gordon M.L., Means B., Brennan R.C., Conklin H.M., Merchant T.E., Vinitsky A., Harman J.L. (2021). Social–emotional functioning in preschool-aged children with cancer: Comparisons between children with brain and non-CNS solid tumors. J. Pediatric Psychol..

[81] Wochos G., Semerjian C., Walsh K.S. (2014). Differences in parent and teacher rating of everyday executive function in pediatric brain tumor survivors. Clin. Neuropsychol..

[82] Wolfe K.R., Walsh K.S., Reynolds N.C., Mitchell F., Reddy A.T., Paltin I., Madan-Swain A. (2013). Executive functions and social skills in survivors of pediatric brain tumor. Child Neuropsychol..

[83] Youn S.H., Ha B., Lee E.H., Park B., Yang S.E., Yu E.S., Kim J.Y. (2022). Neurocognitive and psychological functioning of pediatric brain tumor patients undergoing proton beam therapy for three different tumor types. Pediatric Blood Cancer.

[84] Riehm K.E., Mojtabai R. (2022). Trends in parent-rated emotional symptoms, conduct problems, and hyperactivity/inattention among U.S. children and adolescents, 2004–2019. J. Affect Disord..

[85] Bot M., De Leeuw den Bouter B.J., Adriaanse M.C. (2011). Prevalence of psychosocial problems in Dutch children aged 8–12 years and its association with risk factors and quality of life. Epidemiol. Psychiatr. Sci..

[86] Hudson M.M., Bhatia S., Casillas J., Landier W., Rogers Z.R., Allen C., Harper J., Hord J., Jain J., Warwick A. (2021). Long-term Follow-up Care for Childhood, Adolescent, and Young Adult Cancer Survivors. Pediatrics.

[87] Liptak C., Manley P., Recklitis C.J. (2012). The feasibility of psychosocial screening for adolescent and young adult brain tumor survivors: The value of self-report. J. Cancer Surviv..

[88] Barr R.D., Ferrari A., Ries L., Whelan J., Bleyer W.A. (2016). Cancer in Adolescents and Young Adults: A Narrative Review of the Current Status and a View of the Future. JAMA Pediatr..

[89] Raj S.P., Narad M.E., Salloum R., Platt A., Thompson A., Baum K.T., Wade S.L. (2018). Development of a Web-Based Psychosocial Intervention for Adolescent and Young Adult Survivors of Pediatric Brain Tumor. J. Adolesc. Young Adult Oncol..

[90] Wade S.L., Narad M.E., Moscato E.L., LeBlond E.I., King J.A., Raj S.P., Platt A., Thompson A.N., Baum K.T., Salloum R. (2020). A Survivor’s Journey: Preliminary efficacy of an online problem-solving therapy for survivors of pediatric brain tumor. Pediatr Blood Cancer.

[91] Barrera M., Atenafu E.G., Sung L., Bartels U., Schulte F., Chung J., Cataudella D., Hancock K., Janzen L., Saleh A. (2018). A randomized control intervention trial to improve social skills and quality of life in pediatric brain tumor survivors. Psychooncology.

[92] Conklin H.M., Reddick W.E., Ashford J., Ogg S., Howard S.C., Morris E.B., Brown R., Bonner M., Christensen R., Wu S. (2010). Long-term efficacy of methylphenidate in enhancing attention regulation, social skills, and academic abilities of childhood cancer survivors. J. Clin. Oncol..

[93] Achenbach T.M. (1991). and C. Edelbrock, Child behavior checklist. Burlington (Vt).

[94] Gray S.A.O., Carter A.S., Volkmar F.R. (2013). Adaptive Behavior Assessment System, Second Edition. Encyclopedia of Autism Spectrum Disorders.

[95] Roth R.M., Gioia G.A., Guy S.C., Kenworthy L., Isquith P.K. (2000). Behavior Rating Inventory of Executive Function: BRIEF.

[96] Reynolds C.R. (2010). Behavior assessment system for children. Corsini Encycl. Psychol..

[97] Gresham F.M., Elliott S.N. (2008). Social Skills Improvement System: Rating Scales Manual.

[98] Achenbach T.M. (1991). Manual for the Youth Self-Report and 1991 Profile.

[99] Sparrow S., Cicchetti D., Saulnier C. (2016). Vineland Adaptive Behavior Scales.

[100] Muris P., Meesters C., van den Berg F. (2003). The strengths and difficulties questionnaire (SDQ). Eur. Child Adolesc. Psychiatry.

[101] Gershon R.C., Cella D., A Fox N., Havlik R.J., Hendrie H.C., Wagster M.V. (2010). Assessment of neurological and behavioural function: The NIH Toolbox. Lancet Neurol..

[102] Gresham F.M., Elliott S.N., Service A.G. (1990). Social Skills Rating System Manual.

[103] Constantino J.N., Gruber C. (2005). Social Responsiveness Scale (SRS).

[104] Shahid A., Wilkinson K., Marcu S., Shapiro C.M., Center for Epidemiological Studies Depression Scale for Children (CES-DC) (2011). STOP, THAT and One Hundred Other Sleep Scales.

[105] Harter S. (2012). Self-Perception Profile for Children: Manual and Questionnaires (Grades 3–8).

[106] Pappas D. (2006). ADHD Rating Scale-IV: Checklists, norms, and clinical interpretation. J. Psychoeduc. Assess..

[107] Derogatis L. (2001). BSI-18: Brief Symptom Inventory 18—Administration, Scoring, and Procedures Manual.

[108] Beck A.T., Epstein N., Brown G., Steer R. (1993). Beck Anxiety Inventory Manual.

[109] Beck A.T., Steer R.A., Brown G.K. (1987). Beck Depression Inventory.

[110] Monga S., Birmaher B., Chiappetta L., Brent D., Kaufman J., Bridge J., Cully M. (2000). Screen for child anxiety-related emotional disorders (SCARED): Convergent and divergent validity. Depress. Anxiety.

[111] Beck J.S., Beck A.T., Jolly J.B. (2001). Beck Youth Inventories of Emotional & Social Impairment: Depression Inventory for Youth, Anxiety Inventory for Youth, Anger Inventory for Youth, Disruptive Behavior for Youth, Self-Concept Inventory for Youth: Manual.

[112] Cho S. (2006). A standardization study of the Korean Personality Rating Scale for Children (KPRC). Korean J. Clin. Psychol..

[113] Sandberg M.A. (2018). Neurobehavioral Functioning Inventory. Encyclopedia of Clinical Neuropsychology.

[114] Torki R.N., Doerner E.E., Kaye N.C., Tsutsui E.M., Merrell K.W. (2010). Social Emotional Assets and Resilience Scales: Status and New Developments. Psychology.

