# Supplementary material for: Neurobehavioral Impairment in Pediatric Brain Tumor Survivors: A Meta-Analysis
Source: Cancers (Basel). 2022 Jul 4;14(13):3269. doi: 10.3390/cancers14133269 (PMC9265927; doi:10.3390/cancers14133269)
Supplement: Supplementary file 1 [file cancers-14-03269-s001.zip › Figures S1--S7.pdf]

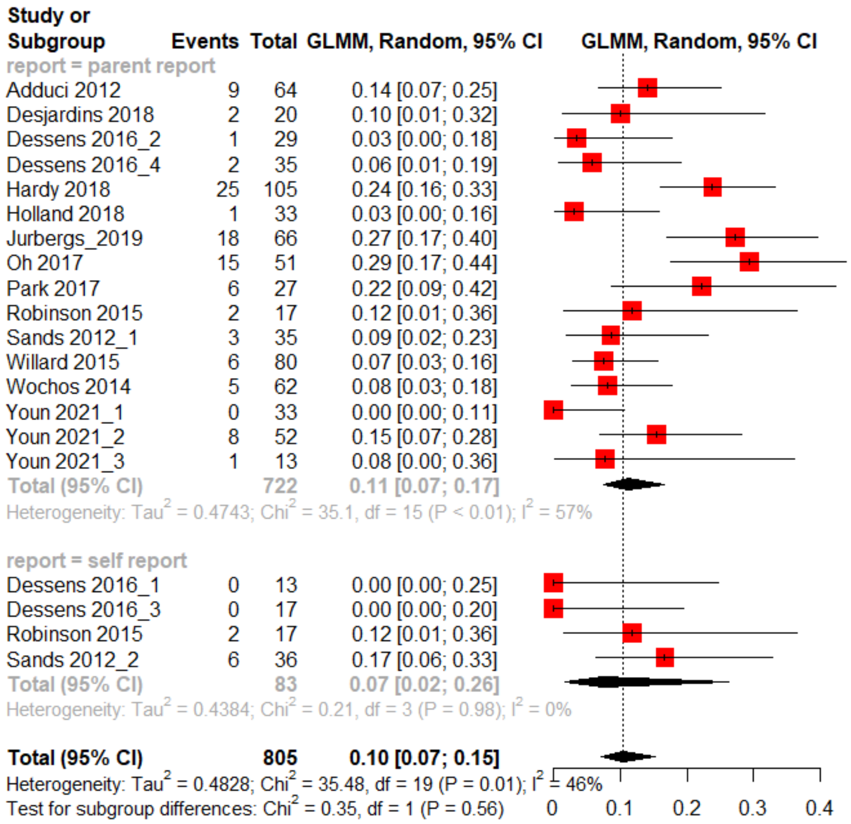

Figure S1a. The absolute risk of pediatric brain tumor survivors having attention problems according to different reporting methods.

Study or Subgroup	TE	SE	Weight	Std. Mean Difference IV, Random, 95% CI
report = parent report				
Adduci 2012	0.71	0.1257	5.6%	0.71 [0.46; 0.96]
Ait Khelifa-Gallois 2015	0.70	0.3606	2.6%	0.70 [-0.01; 1.41]
Alias 2020	0.70	0.1612	5.0%	0.70 [0.38; 1.02]
De Vries 2018	0.17	0.1175	5.7%	0.17 [-0.06; 0.40]
Desjardins 2018	0.85	0.2236	4.2%	0.85 [0.41; 1.29]
Heitzer 2019	1.16	0.2186	4.2%	1.16 [0.73; 1.59]
Holland 2018	0.08	0.1844	4.7%	0.08 [-0.28; 0.44]
Jurbergs_2019	0.63	0.1112	5.7%	0.63 [0.41; 0.85]
Oh 2017	0.14	0.1404	5.3%	0.14 [-0.14; 0.41]
Park 2017	0.38	0.6091	1.2%	0.38 [-0.82; 1.57]
Puhr 2021_1	0.65	0.1905	4.6%	0.65 [0.28; 1.02]
Robinson 2015	0.74	0.3667	2.6%	0.74 [0.02; 1.46]
Sands 2012_1	-0.21	0.1670	5.0%	-0.21 [-0.54; 0.12]
Willard 2015	0.40	0.1122	5.7%	0.40 [0.18; 0.62]
willard 2017	1.30	0.3701	2.5%	1.30 [0.57; 2.03]
Wochos 2014	0.00	0.1918	4.6%	0.00 [-0.38; 0.38]
Youn 2021_1	0.05	0.1732	4.9%	0.05 [-0.29; 0.39]
Youn 2021_2	0.55	0.1389	5.4%	0.55 [0.28; 0.82]
Youn 2021_3	0.88	0.2775	3.5%	0.88 [0.34; 1.42]
Raghubar 2018	0.29	0.1860	4.7%	0.29 [-0.08; 0.65]
**Total (95% CI)**			**87.7%**	**0.47 [0.30; 0.64]**
Heterogeneity: $Tau^2 = 0.1054$; $Chi^2 = 74.93$, df = 19 ($P < 0.01$); $I^2 = 75\%$				
report = self report				
Robinson 2015	0.37	0.3503	2.7%	0.37 [-0.32; 1.05]
Puhr 2021_2	0.44	0.1879	4.7%	0.44 [0.07; 0.81]
Sands 2012_2	0.53	0.1670	5.0%	0.53 [0.21; 0.86]
**Total (95% CI)**			**12.3%**	**0.48 [0.25; 0.71]**
Heterogeneity: $Tau^2 = 0$; $Chi^2 = 0.25$, df = 2 ($P = 0.88$); $I^2 = 0\%$				
**Total (95% CI)**			**100.0%**	**0.47 [0.32; 0.61]**
Heterogeneity: $Tau^2 = 0.0855$; $Chi^2 = 75.34$, df = 22 ($P < 0.01$); $I^2 = 71\%$				
Test for subgroup differences: $Chi^2 = 0.00$, df = 1 ($P = 0.97$)				

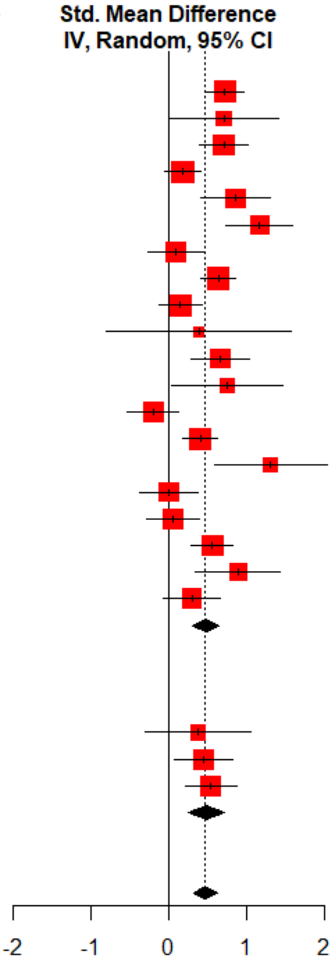

Figure S1b. The standard mean difference of pediatric brain tumor survivors having attention problems according to different reporting methods, as compared to population norm or healthy controls.

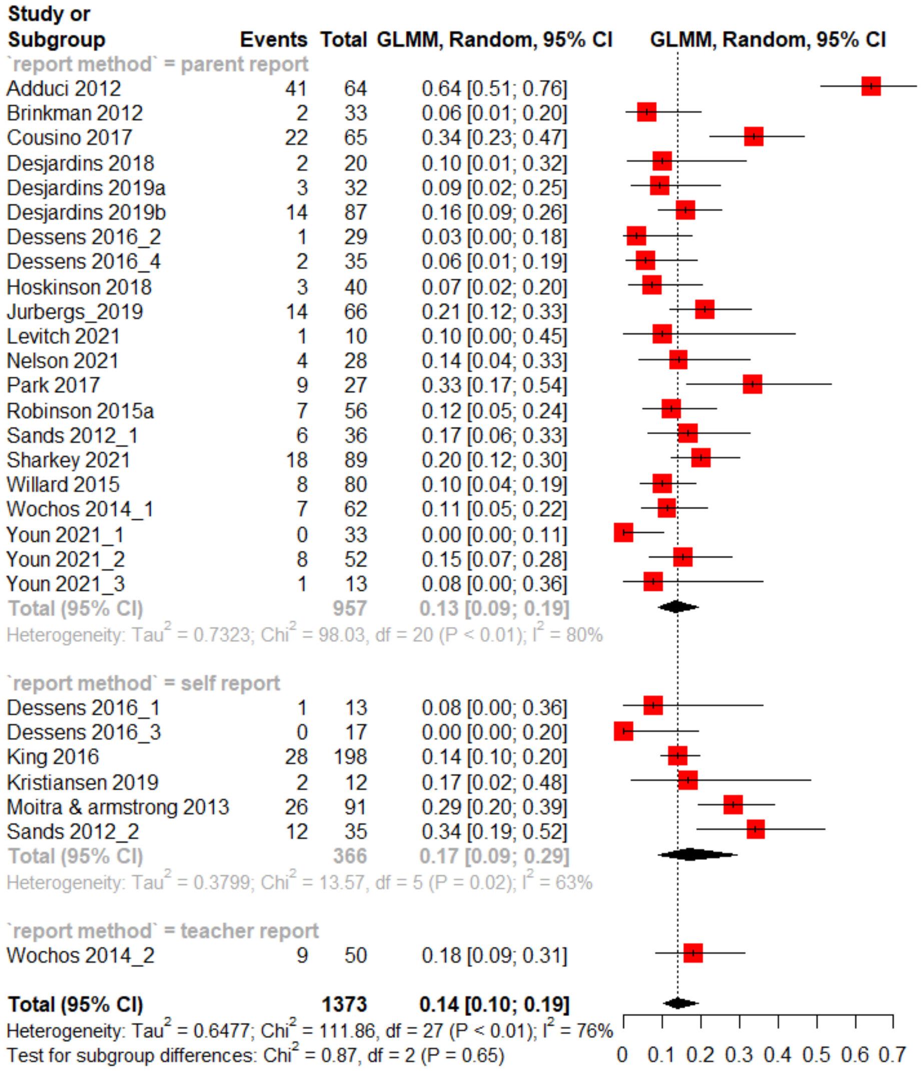

Figure S1c. The absolute risk of pediatric brain tumor survivors having emotional difficulities according to different reporting methods.

Study or Subgroup	TE	SE	Weight	Std. Mean Difference IV, Random, 95% CI
`report method` = parent report				
Aarsen 2014_1	0.79	0.2887	1.7%	0.79 [0.23; 1.36]
Adduci 2012	1.08	0.1225	3.9%	1.08 [0.84; 1.32]
Ait Khelifa-Gallois 2015	0.78	0.3680	1.2%	0.78 [0.06; 1.50]
Alias 2020	0.72	0.2369	2.2%	0.72 [0.25; 1.18]
Cousino 2017	0.21	0.1245	3.9%	0.21 [-0.03; 0.45]
De Vries 2018	0.47	0.1175	4.0%	0.47 [0.24; 0.70]
Desjardins 2018	0.85	0.2236	2.3%	0.85 [0.41; 1.29]
Desjardins 2019a	0.02	0.1772	3.0%	0.02 [-0.33; 0.37]
Desjardins 2019b	0.40	0.1288	3.8%	0.40 [0.15; 0.65]
Emond 2016_2	0.97	0.2828	1.7%	0.97 [0.42; 1.53]
Heitzer 2019	0.43	0.2296	2.3%	0.43 [-0.02; 0.88]
Hoskinson 2018	0.04	0.1584	3.3%	0.04 [-0.27; 0.35]
Jurbergs_2019	0.40	0.1237	3.9%	0.40 [0.16; 0.64]
Levitch 2021	0.33	0.3162	1.5%	0.33 [-0.29; 0.95]
Oh 2017	0.15	0.1404	3.6%	0.15 [-0.13; 0.42]
Park 2017	0.49	0.1926	2.7%	0.49 [0.11; 0.87]
Puhr 2021	0.26	0.1865	2.8%	0.26 [-0.11; 0.62]
Robinson 2015a	0.25	0.1703	3.1%	0.25 [-0.08; 0.59]
Robinson 2015b_1	0.42	0.3583	1.2%	0.42 [-0.28; 1.13]
Sands 2012_1	0.12	0.1694	3.1%	0.12 [-0.21; 0.45]
Sharkey 2021	0.45	0.1063	4.2%	0.45 [0.24; 0.66]
Wier 2019	0.86	0.4215	0.9%	0.86 [0.04; 1.69]
Willard 2015	0.19	0.1122	4.1%	0.19 [-0.03; 0.41]
willard 2021	0.32	0.2088	2.5%	0.32 [-0.09; 0.73]
Wochos 2014_1	0.34	0.1146	4.1%	0.34 [0.11; 0.56]
Youn 2021_1	0.11	0.1732	3.0%	0.11 [-0.23; 0.45]
Youn 2021_2	0.39	0.1389	3.6%	0.39 [0.12; 0.66]
Youn 2021_3	0.53	0.2775	1.8%	0.53 [-0.01; 1.07]
Total (95% CI)			79.6%	0.40 [0.30; 0.50]
Heterogeneity: $Tau^2 = 0.0415$; $Chi^2 = 71.95$, df = 27 ($P < 0.01$); $I^2 = 62\%$				
`report method` = self report				
Aarsen 2014_2	0.89	0.2887	1.7%	0.89 [0.32; 1.46]
Cheung 2019	0.64	0.1162	4.0%	0.64 [0.41; 0.87]
Emond 2016_1	0.01	0.2443	2.1%	0.01 [-0.46; 0.49]
King 2016	0.38	0.1844	2.9%	0.38 [0.02; 0.74]
Puhr 2021_2	0.69	0.1910	2.8%	0.69 [0.31; 1.06]
Robinson 2015b_2	0.41	0.3582	1.2%	0.41 [-0.29; 1.11]
Sands 2012_2	0.54	0.1670	3.1%	0.54 [0.22; 0.87]
Total (95% CI)			17.8%	0.53 [0.35; 0.71]
Heterogeneity: $Tau^2 = 0.0189$; $Chi^2 = 8.32$, df = 6 ($P = 0.22$); $I^2 = 28\%$				
`report method` = teacher report				
Emond 2016_3	0.12	0.3844	1.1%	0.12 [-0.64; 0.87]
Wochos 2014_2	0.68	0.3143	1.5%	0.68 [0.06; 1.29]
Total (95% CI)			2.6%	0.44 [-0.10; 0.98]
Heterogeneity: $Tau^2 = 0.0340$; $Chi^2 = 1.28$, df = 1 ($P = 0.26$); $I^2 = 22\%$				
**Total (95% CI)**			**100.0%**	**0.42 [0.34; 0.51]**
Heterogeneity: $Tau^2 = 0.0363$; $Chi^2 = 85.83$, df = 36 ($P < 0.01$); $I^2 = 58\%$				
Test for subgroup differences: $Chi^2 = 1.44$, df = 2 ($P = 0.49$)				

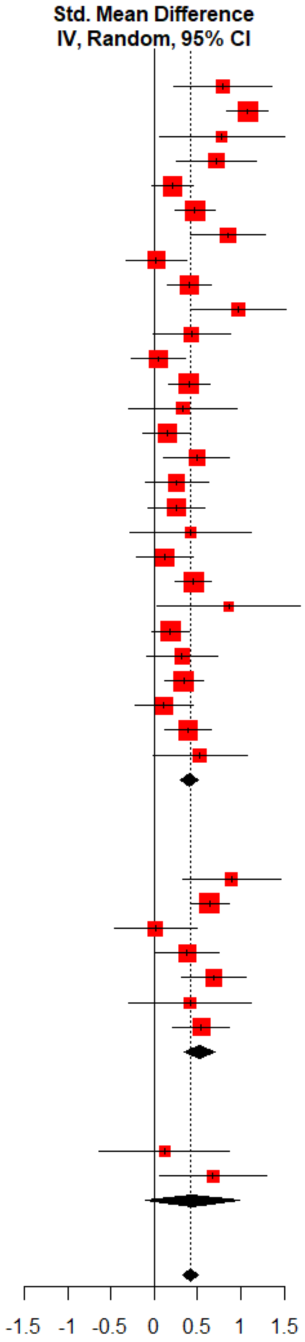

Figure S1d. The standard mean difference of pediatric brain tumor survivors having emotional difficulities according to different reporting methods, as compared to population norm or healthy controls.

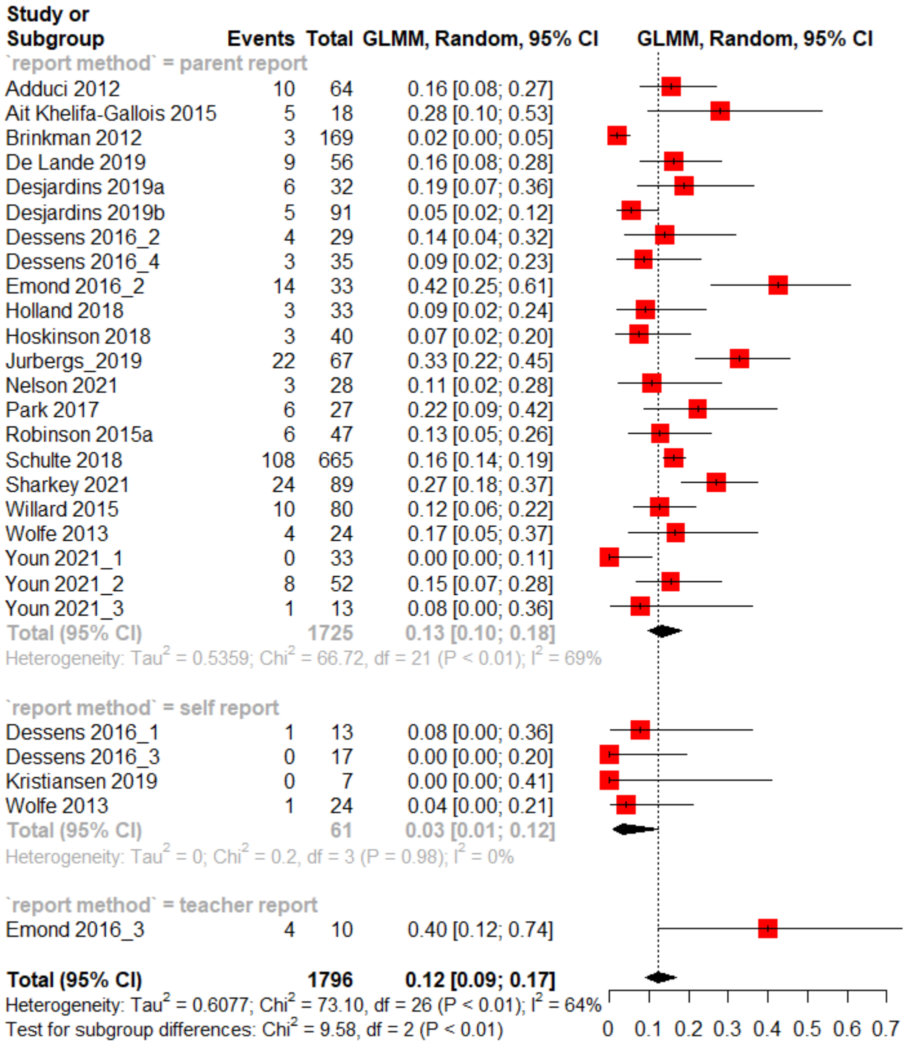

Figure S1e. The absolute risk of pediatric brain tumor survivors having psychosocial problems according to different reporting methods.

Study or Subgroup	TE	SE	Weight	Std. Mean Difference IV, Random, 95% CI
`report method` = parent report				
Adduci 2012	1.00	0.1257	2.9%	1.00 [0.75; 1.24]
Ait Khelifa-Gallois 2015	0.00	0.3543	1.5%	0.00 [-0.69; 0.69]
Albee 2022	-0.16	0.1404	2.8%	-0.16 [-0.44; 0.12]
Alias 2020	0.85	0.1612	2.7%	0.85 [0.53; 1.17]
Brinkman 2012	0.36	0.2786	1.9%	0.36 [-0.19; 0.90]
de Lande 2019	0.51	0.2000	2.4%	0.51 [0.11; 0.90]
Desjardins 2019a	0.14	0.1772	2.6%	0.14 [-0.21; 0.49]
Desjardins 2019b	0.04	0.1049	3.1%	0.04 [-0.17; 0.25]
Desjardins 2021	0.66	0.1965	2.4%	0.66 [0.27; 1.04]
Emond 2016_2	0.61	0.2739	1.9%	0.61 [0.07; 1.14]
Gordon 2022_2	0.39	0.1288	2.9%	0.39 [0.14; 0.64]
Hocking 2017	0.10	0.1670	2.6%	0.10 [-0.23; 0.42]
Hocking 2021	1.27	0.2236	2.2%	1.27 [0.83; 1.71]
Holland 2018	0.88	0.1844	2.5%	0.88 [0.51; 1.24]
Hoskinson 2018	0.02	0.1584	2.7%	0.02 [-0.29; 0.33]
Jurbergs_2019	0.79	0.1229	2.9%	0.79 [0.55; 1.03]
Kok 2020_1	0.24	0.2447	2.1%	0.24 [-0.23; 0.72]
Liang 2013	0.53	0.1342	2.9%	0.53 [0.27; 0.79]
Oh 2017	0.14	0.1404	2.8%	0.14 [-0.13; 0.42]
Park 2017	0.61	0.1924	2.5%	0.61 [0.23; 0.98]
Puhr 2021	0.84	0.1658	2.7%	0.84 [0.51; 1.16]
Raghbar 2019	0.97	0.2298	2.2%	0.97 [0.52; 1.42]
Robinson 2015a	0.08	0.1342	2.9%	0.08 [-0.19; 0.34]
Robinson 2015b_2	1.04	0.3785	1.4%	1.04 [0.30; 1.78]
Sharkey 2021	0.85	0.1063	3.1%	0.85 [0.65; 1.06]
Willard 2015	0.36	0.1122	3.0%	0.36 [0.14; 0.58]
Willard 2017	1.16	0.3661	1.4%	1.16 [0.44; 1.87]
Willard 2019_2	0.65	0.2238	2.2%	0.65 [0.22; 1.09]
Willard 2021	0.61	0.2088	2.3%	0.61 [0.20; 1.02]
Wolfe 2013	0.05	0.1926	2.5%	0.05 [-0.33; 0.42]
Youn 2021_1	0.49	0.1732	2.6%	0.49 [0.15; 0.83]
Youn 2021_2	0.46	0.1378	2.8%	0.46 [0.19; 0.73]
Youn 2021_3	0.26	0.2775	1.9%	0.26 [-0.28; 0.80]
Total (95% CI)			81.3%	0.49 [0.37; 0.62]
Heterogeneity: $Tau^2 = 0.0997$; $Chi^2 = 156.32$, df = 32 ($P < 0.01$); $I^2 = 80\%$				
`report method` = self report				
Emond 2016_1	-0.17	0.2466	2.1%	-0.17 [-0.65; 0.31]
Gordon 2022_1	0.41	0.1293	2.9%	0.41 [0.16; 0.66]
Puhr 2021_2	0.50	0.1887	2.5%	0.50 [0.13; 0.87]
Robinson 2015b_1	0.61	0.3629	1.4%	0.61 [-0.10; 1.33]
Willard 2019_1	0.75	0.2238	2.2%	0.75 [0.31; 1.19]
Wolfe 2013	-0.15	0.1926	2.5%	-0.15 [-0.53; 0.22]
Total (95% CI)			13.6%	0.31 [0.00; 0.62]
Heterogeneity: $Tau^2 = 0.1007$; $Chi^2 = 15.69$, df = 5 ($P < 0.01$); $I^2 = 68\%$				
`report method` = teacher report				
Emond 2016_3	0.92	0.3924	1.3%	0.92 [0.15; 1.69]
Kok 2020_2	0.23	0.3385	1.5%	0.23 [-0.43; 0.89]
Willard 2019_3	0.67	0.2238	2.2%	0.67 [0.23; 1.11]
Total (95% CI)			5.1%	0.61 [0.28; 0.94]
Heterogeneity: $Tau^2 = 0$; $Chi^2 = 1.95$, df = 2 ($P = 0.38$); $I^2 = 0\%$				
**Total (95% CI)**			**100.0%**	**0.47 [0.36; 0.59]**
Heterogeneity: $Tau^2 = 0.0946$; $Chi^2 = 177.76$, df = 41 ($P < 0.01$); $I^2 = 77\%$				
Test for subgroup differences: $Chi^2 = 1.74$, df = 2 ($P = 0.42$)				

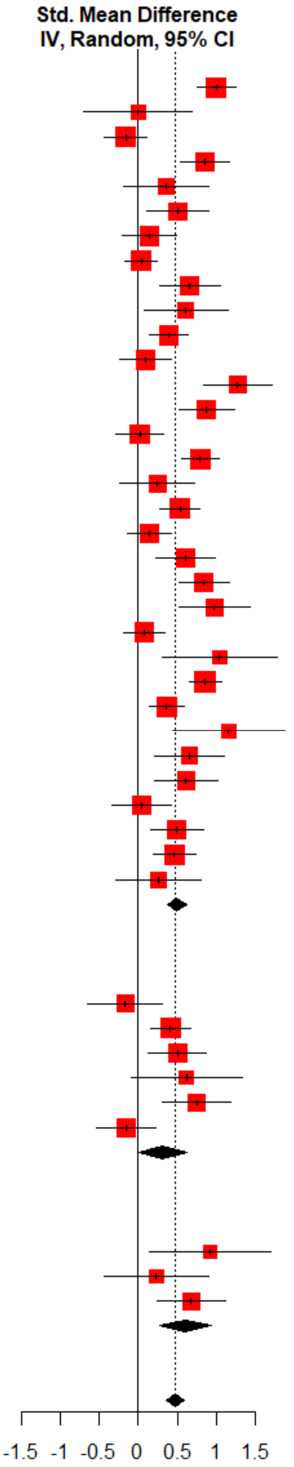

Figure S1f. The standard mean difference of pediatric brain tumor survivors having psychosocial problems according to different reporting methods, as compared to population norm or healthy controls.

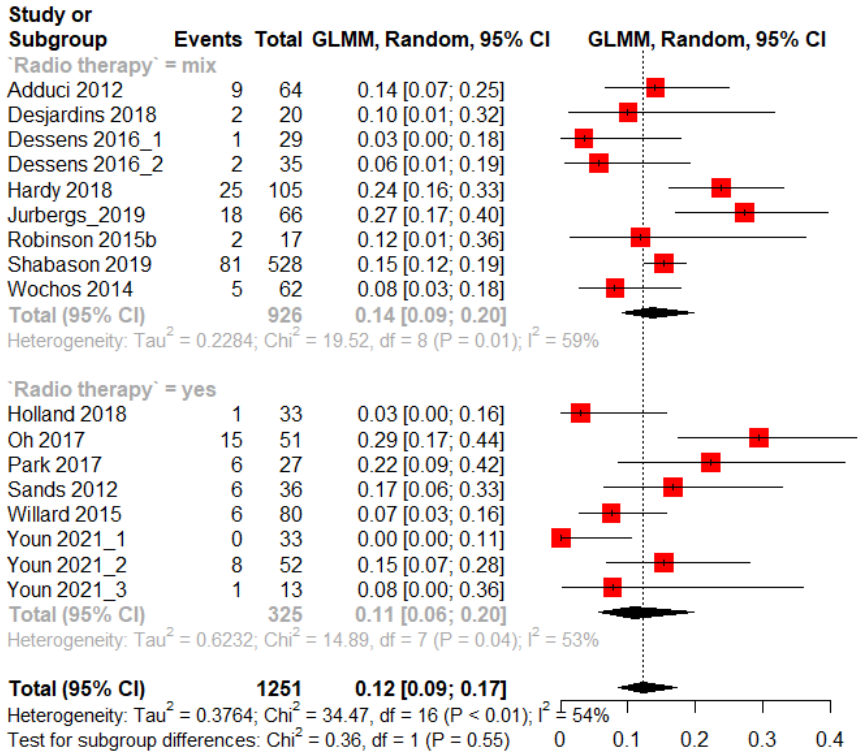

(a) Absolute risk of attention problems

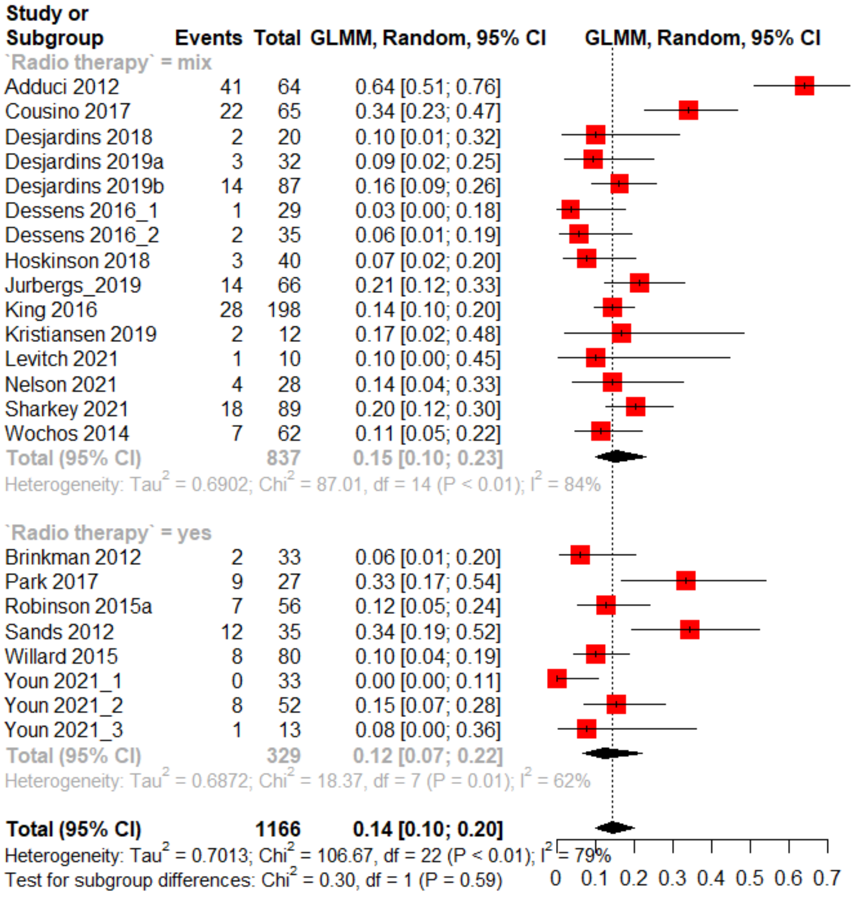

(b) Absolute risk of emotional difficulties

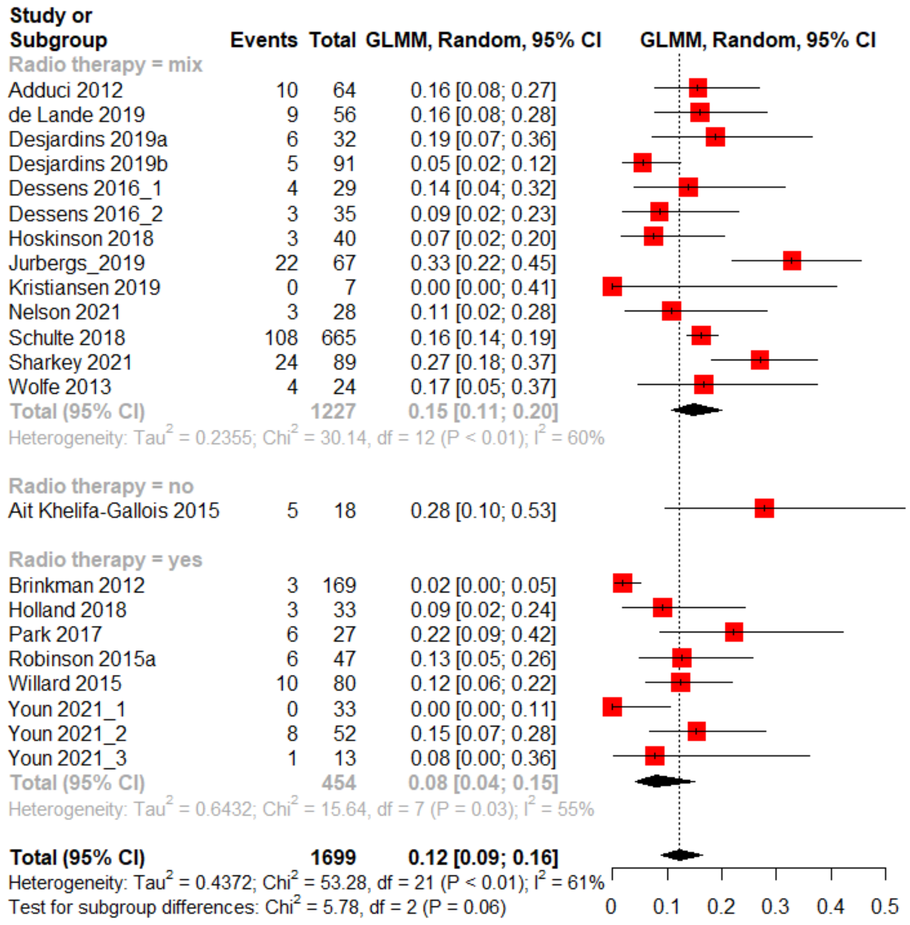

(c) Absolute risk of psychosocial problems

Figure S2. (a-c)Absolute risk of neurobehavioral impairment in paediatric brain tumor survivors by different radio therapy status.

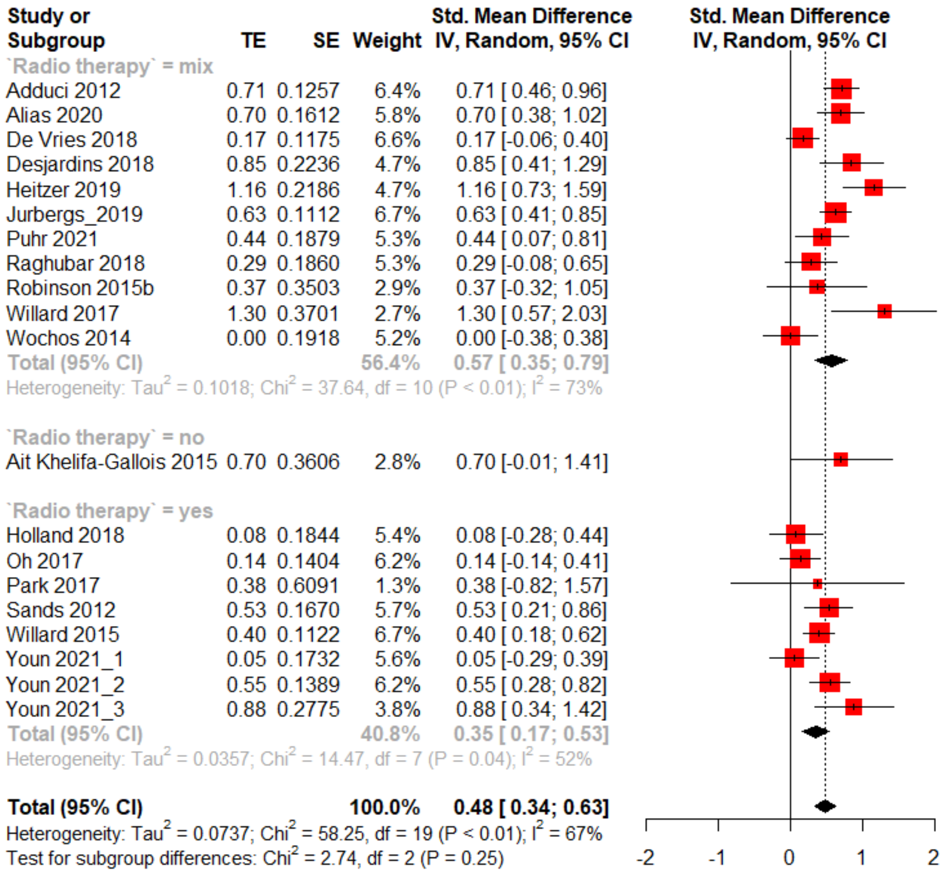

(a) Standard mean difference of attention problems

Study or Subgroup	TE	SE	Weight	Std. Mean Difference IV, Random, 95% CI
`Radio therapy` = no				
Aarsen 2014	0.89	0.2887	1.8%	0.89 [0.32; 1.46]
Ait Khelifa-Gallois 2015	0.78	0.3680	1.3%	0.78 [0.06; 1.50]
Total (95% CI)			3.1%	0.85 [0.40; 1.29]
Heterogeneity: Tau^2 = 0; Chi^2 = 0.06, df = 1 (P = 0.81); I^2 = 0%				
`Radio therapy` = mix				
Adduci 2012	1.08	0.1225	4.3%	1.08 [0.84; 1.32]
Alias 2020	0.72	0.2369	2.4%	0.72 [0.25; 1.18]
Cheung 2019	0.64	0.1162	4.4%	0.64 [0.41; 0.87]
Cousino 2017	0.21	0.1245	4.2%	0.21 [-0.03; 0.45]
De Vries 2018	0.47	0.1175	4.4%	0.47 [0.24; 0.70]
Desjardins 2018	0.85	0.2236	2.6%	0.85 [0.41; 1.29]
Desjardins 2019a	0.02	0.1772	3.3%	0.02 [-0.33; 0.37]
Desjardins 2019b	0.40	0.1288	4.2%	0.40 [0.15; 0.65]
Emond 2016	0.01	0.2443	2.3%	0.01 [-0.46; 0.49]
Heitzer 2019	0.43	0.2296	2.5%	0.43 [-0.02; 0.88]
Hoskinson 2018	0.04	0.1584	3.6%	0.04 [-0.27; 0.35]
Jurbergs_2019	0.40	0.1237	4.3%	0.40 [0.16; 0.64]
King 2016	0.38	0.1844	3.1%	0.38 [0.02; 0.74]
Levitch 2021	0.33	0.3162	1.6%	0.33 [-0.29; 0.95]
Puhr 2021	0.69	0.1910	3.0%	0.69 [0.31; 1.06]
Robinson 2015b	0.41	0.3582	1.3%	0.41 [-0.29; 1.11]
Sharkey 2021	0.45	0.1063	4.6%	0.45 [0.24; 0.66]
Wier 2019	0.86	0.4215	1.0%	0.86 [0.04; 1.69]
Willard 2021	0.32	0.2088	2.8%	0.32 [-0.09; 0.73]
Wochos 2014	0.34	0.1146	4.4%	0.34 [0.11; 0.56]
Total (95% CI)			64.2%	0.45 [0.32; 0.57]
Heterogeneity: Tau^2 = 0.0461; Chi^2 = 57.39, df = 19 (P < 0.01); I^2 = 67%				
`Radio therapy` = yes				
Brinkman 2012	0.60	0.0775	5.2%	0.60 [0.45; 0.75]
Oh 2017	0.15	0.1404	3.9%	0.15 [-0.13; 0.42]
Park 2017	0.49	0.1926	3.0%	0.49 [0.11; 0.87]
Robinson 2015a	0.25	0.1703	3.4%	0.25 [-0.08; 0.59]
Sands 2012	0.54	0.1670	3.4%	0.54 [0.22; 0.87]
Willard 2015	0.19	0.1122	4.5%	0.19 [-0.03; 0.41]
Youn 2021_1	0.11	0.1732	3.3%	0.11 [-0.23; 0.45]
Youn 2021_2	0.39	0.1389	4.0%	0.39 [0.12; 0.66]
Youn 2021_3	0.53	0.2775	1.9%	0.53 [-0.01; 1.07]
Total (95% CI)			32.6%	0.36 [0.23; 0.49]
Heterogeneity: Tau^2 = 0.0158; Chi^2 = 18.2, df = 8 (P = 0.02); I^2 = 56%				
**Total (95% CI)**			**100.0%**	**0.43 [0.34; 0.52]**
Heterogeneity: Tau^2 = 0.0367; Chi^2 = 80.08, df = 30 (P < 0.01); I^2 = 63%				
Test for subgroup differences: Chi^2 = 4.52, df = 2 (P = 0.10)				

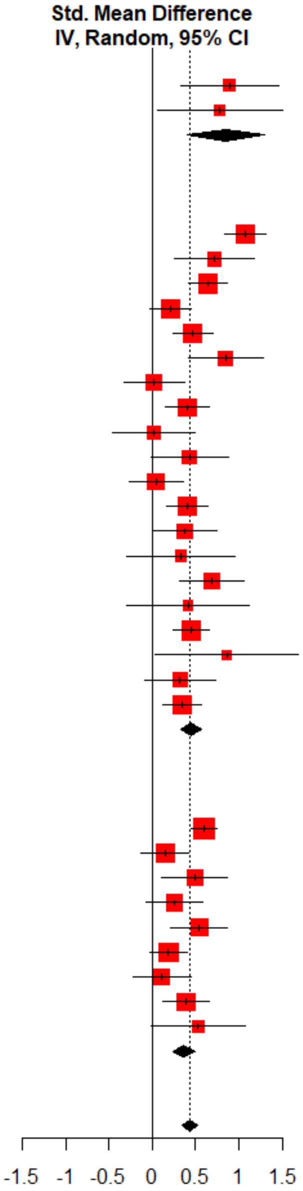

(b) Standard mean difference of emotional difficulties

Study or Subgroup	TE	SE	Weight	Std. Mean Difference IV, Random, 95% CI
Radio therapy = mix				
Adduci 2012	1.00	0.1257	3.6%	1.00 [0.75; 1.24]
Albee 2022	-0.16	0.1404	3.5%	-0.16 [-0.44; 0.12]
Alias 2020	0.85	0.1612	3.3%	0.85 [0.53; 1.17]
de Lande 2019	0.51	0.2000	3.0%	0.51 [0.11; 0.90]
Desjardins 2019a	0.14	0.1772	3.2%	0.14 [-0.21; 0.49]
Desjardins 2019b	0.04	0.1049	3.7%	0.04 [-0.17; 0.25]
Desjardins 2021	0.66	0.1965	3.0%	0.66 [0.27; 1.04]
Emond 2016	-0.17	0.2466	2.6%	-0.17 [-0.65; 0.31]
Gordon 2022	0.39	0.1288	3.5%	0.39 [0.14; 0.64]
Hocking 2017	0.10	0.1670	3.2%	0.10 [-0.23; 0.42]
Hocking 2021	1.27	0.2236	2.8%	1.27 [0.83; 1.71]
Hoskinson 2018	0.02	0.1584	3.3%	0.02 [-0.29; 0.33]
Jurbergs_2019	0.79	0.1229	3.6%	0.79 [0.55; 1.03]
Puhr 2021	0.50	0.1887	3.1%	0.50 [0.13; 0.87]
Robinson 2015b	0.61	0.3629	1.8%	0.61 [-0.10; 1.33]
Sharkey 2021	0.85	0.1063	3.7%	0.85 [0.65; 1.06]
Willard 2017	1.16	0.3661	1.8%	1.16 [0.44; 1.87]
Willard 2019	0.75	0.2238	2.8%	0.75 [0.31; 1.19]
Willard 2021	0.61	0.2088	2.9%	0.61 [0.20; 1.02]
Wolfe 2013	0.05	0.1926	3.0%	0.05 [-0.33; 0.42]
Total (95% CI)			61.2%	0.48 [0.30; 0.67]
Heterogeneity: $Tau^2 = 0.1459$; $Chi^2 = 127.83$, df = 19 ($P < 0.01$); $I^2 = 85\%$				
Radio therapy = no				
Ait Khelifa-Gallois 2015	0.00	0.3543	1.8%	0.00 [-0.69; 0.69]
Kok 2020	0.24	0.2447	2.6%	0.24 [-0.23; 0.72]
Total (95% CI)			4.4%	0.17 [-0.23; 0.56]
Heterogeneity: $Tau^2 = 0$; $Chi^2 = 0.32$, df = 1 ($P = 0.57$); $I^2 = 0\%$				
Radio therapy = yes				
Brinkman 2012	0.36	0.2786	2.3%	0.36 [-0.19; 0.90]
Holland 2018	0.88	0.1844	3.1%	0.88 [0.51; 1.24]
Liang 2013	0.53	0.1342	3.5%	0.53 [0.27; 0.79]
Oh 2017	0.14	0.1404	3.5%	0.14 [-0.13; 0.42]
Park 2017	0.61	0.1924	3.0%	0.61 [0.23; 0.98]
Raghbar 2019	0.97	0.2298	2.7%	0.97 [0.52; 1.42]
Robinson 2015a	0.08	0.1342	3.5%	0.08 [-0.19; 0.34]
Willard 2015	0.36	0.1122	3.7%	0.36 [0.14; 0.58]
Youn 2021_1	0.49	0.1732	3.2%	0.49 [0.15; 0.83]
Youn 2021_2	0.46	0.1378	3.5%	0.46 [0.19; 0.73]
Youn 2021_3	0.26	0.2775	2.3%	0.26 [-0.28; 0.80]
Total (95% CI)			34.3%	0.45 [0.29; 0.61]
Heterogeneity: $Tau^2 = 0.0428$; $Chi^2 = 24.96$, df = 10 ($P < 0.01$); $I^2 = 60\%$				
**Total (95% CI)**			**100.0%**	**0.46 [0.33; 0.58]**
Heterogeneity: $Tau^2 = 0.1018$; $Chi^2 = 155.91$, df = 32 ($P < 0.01$); $I^2 = 79\%$				
Test for subgroup differences: $Chi^2 = 2.08$, df = 2 ($P = 0.35$)				

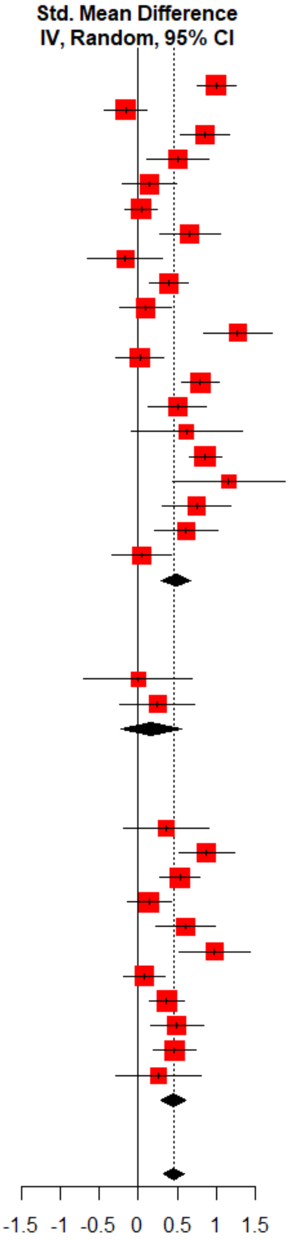

(c) Standard mean difference of psychosocial problems

Figure S3. (a-c) Standard mean difference of neurobehavioral impairment in paediatric brain tumor survivors compared to healthy controls and population norm by different radio therapy status.

Study or Subgroup	TE	SE	Weight	Std. Mean Difference IV, Random, 95% CI
RT = n				
Aarsen 2014 emotional problems	0.89	0.2887	1.7%	0.89 [0.32; 1.46]
Ait Khelifa-Gallois 2015 attention problems	0.70	0.3606	1.2%	0.70 [-0.01; 1.41]
Ait Khelifa-Gallois 2015 emotional problems	0.78	0.3680	1.1%	0.78 [0.06; 1.50]
Ait Khelifa-Gallois 2015 social problems	0.00	0.3543	1.2%	0.00 [-0.69; 0.69]
Kok 2020 social problems	0.24	0.2447	2.1%	0.24 [-0.23; 0.72]
Total (95% CI)			7.4%	0.51 [0.18; 0.84]
Heterogeneity: Tau^2 = 0.0426; Chi^2 = 5.79, df = 4 (P = 0.22); I^2 = 31%				
RT = y				
Brinkman 2012 emotional problems	0.60	0.0775	5.5%	0.60 [0.45; 0.75]
Brinkman 2012 social problems	0.36	0.2786	1.8%	0.36 [-0.19; 0.90]
Holland 2018 attention problems	0.08	0.1844	3.0%	0.08 [-0.28; 0.44]
Holland 2018 social problems	0.88	0.1844	3.0%	0.88 [0.51; 1.24]
Liang 2013 social problems	0.53	0.1342	4.1%	0.53 [0.27; 0.79]
Oh 2017 attention problems	0.14	0.1404	3.9%	0.14 [-0.14; 0.41]
Oh 2017 emotional problems	0.15	0.1404	3.9%	0.15 [-0.13; 0.42]
Oh 2017 social problems	0.14	0.1404	3.9%	0.14 [-0.13; 0.42]
Park 2017 attention problems	0.38	0.6091	0.5%	0.38 [-0.82; 1.57]
Park 2017 emotional problems	0.49	0.1926	2.9%	0.49 [0.11; 0.87]
Park 2017 social problems	0.61	0.1924	2.9%	0.61 [0.23; 0.98]
Raghbar 2019 social problems	0.97	0.2298	2.3%	0.97 [0.52; 1.42]
Robinson 2015a emotional problems	0.25	0.1703	3.3%	0.25 [-0.08; 0.59]
Robinson 2015a social problems	0.08	0.1342	4.1%	0.08 [-0.19; 0.34]
Sands 2012 attention problems	0.53	0.1670	3.4%	0.53 [0.21; 0.86]
Sands 2012 emotional problems	0.54	0.1670	3.4%	0.54 [0.22; 0.87]
Willard 2015 attention problems	0.40	0.1122	4.6%	0.40 [0.18; 0.62]
Willard 2015 emotional problems	0.19	0.1122	4.6%	0.19 [-0.03; 0.41]
Willard 2015 social problems	0.36	0.1122	4.6%	0.36 [0.14; 0.58]
Youn 2021_1 attention problems	0.05	0.1732	3.2%	0.05 [-0.29; 0.39]
Youn 2021_1 emotional problems	0.11	0.1732	3.2%	0.11 [-0.23; 0.45]
Youn 2021_1 social problems	0.49	0.1732	3.2%	0.49 [0.15; 0.83]
Youn 2021_2 attention problems	0.55	0.1389	4.0%	0.55 [0.28; 0.82]
Youn 2021_2 emotional problems	0.39	0.1389	4.0%	0.39 [0.12; 0.66]
Youn 2021_2 social problems	0.46	0.1378	4.0%	0.46 [0.19; 0.73]
Youn 2021_3 attention problems	0.88	0.2775	1.8%	0.88 [0.34; 1.42]
Youn 2021_3 emotional problems	0.53	0.2775	1.8%	0.53 [-0.01; 1.07]
Youn 2021_3 social problems	0.26	0.2775	1.8%	0.26 [-0.28; 0.80]
Total (95% CI)			92.6%	0.39 [0.30; 0.48]
Heterogeneity: Tau^2 = 0.0282; Chi^2 = 58.51, df = 27 (P < 0.01); I^2 = 54%				
**Total (95% CI)**			**100.0%**	**0.40 [0.31; 0.48]**
Heterogeneity: Tau^2 = 0.0282; Chi^2 = 64.90, df = 32 (P < 0.01); I^2 = 51%				
Test for subgroup differences: Chi^2 = 0.48, df = 1 (P = 0.49)				

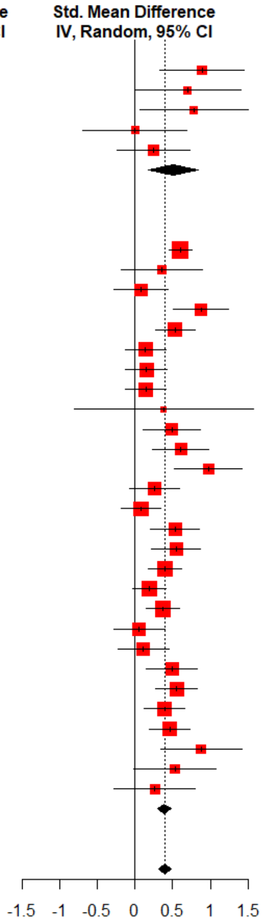

Figure S4. The pooled standard mean difference of pediatric brain tumor survivors having neurobehavioral impairment in patients with or without radiotherapy.

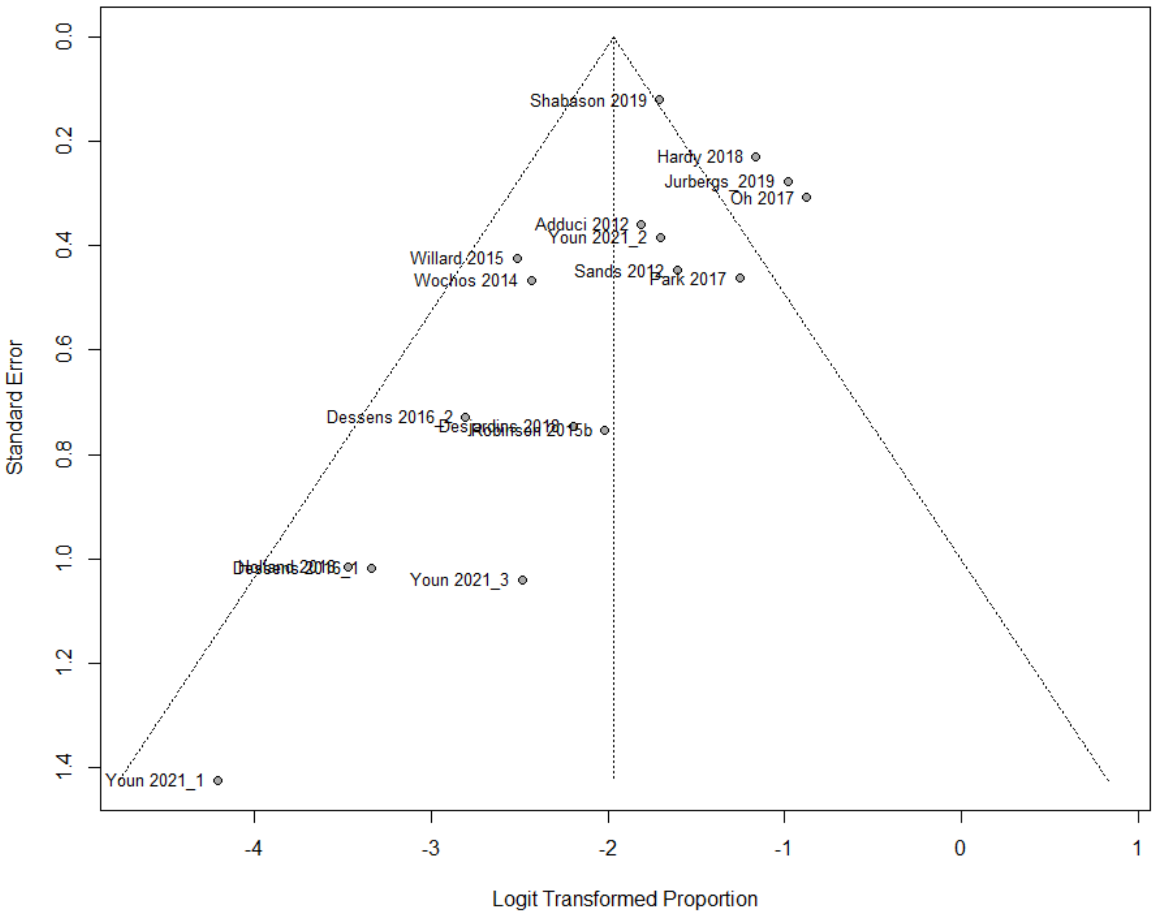

(a) Attention problems – absolute risk

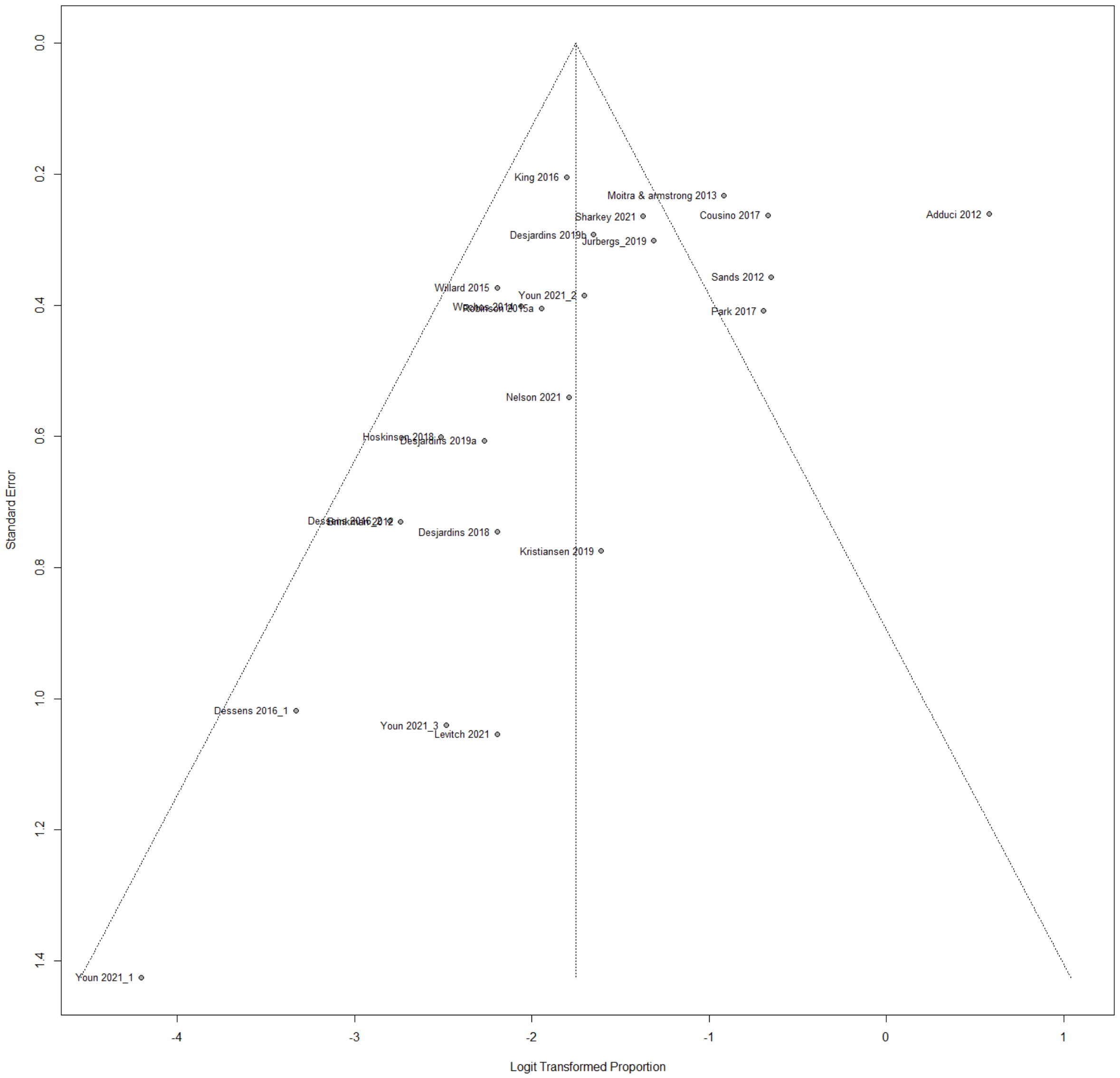

(b) Emotional difficulties – absolute risk

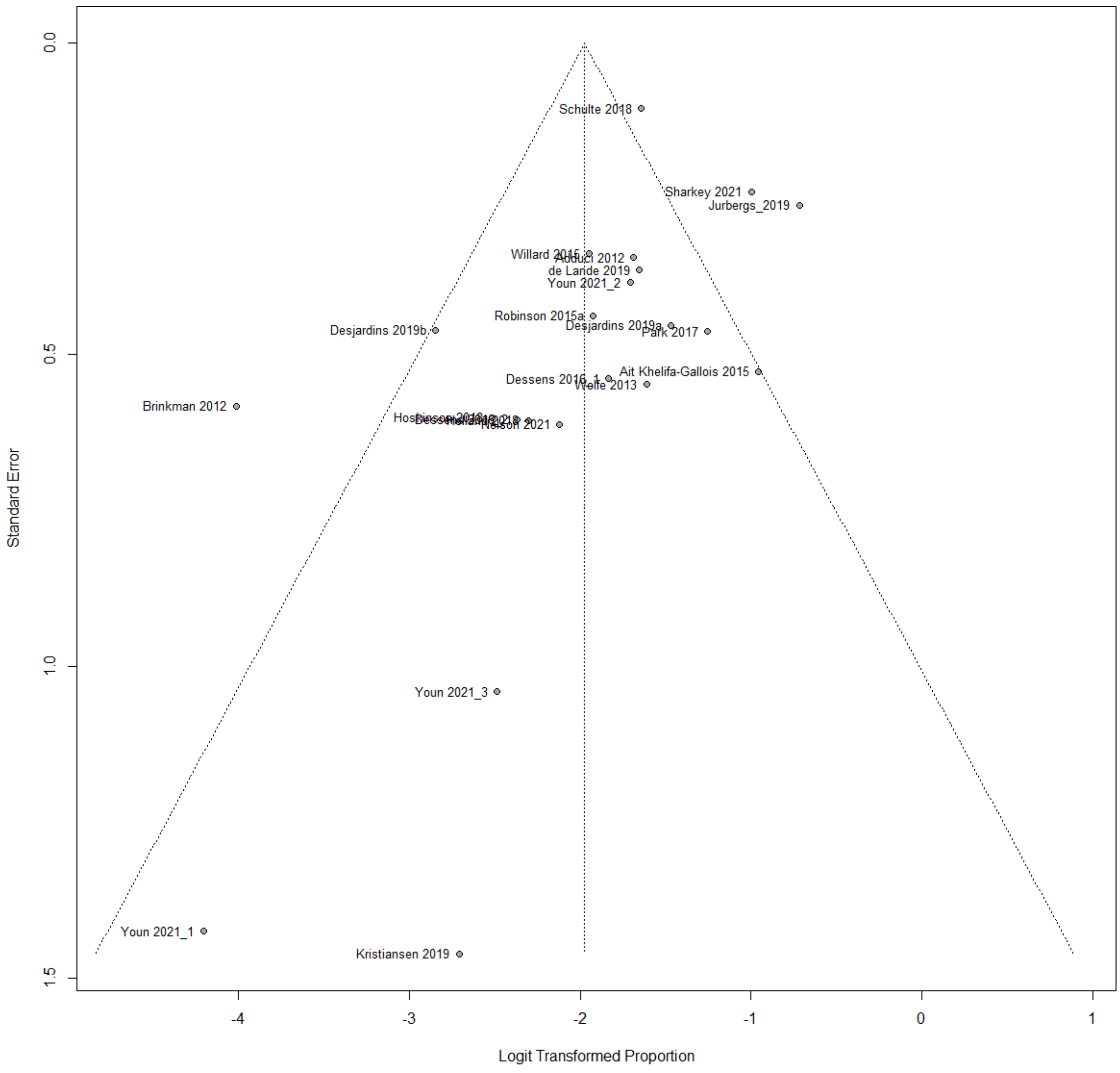

(c) Psychosocial problems – absolute risk

Figure S5. (a-c) Funnel plot of logit transformation of neurobehavioral impairment absolute risk in paediatric brain tumor survivors.

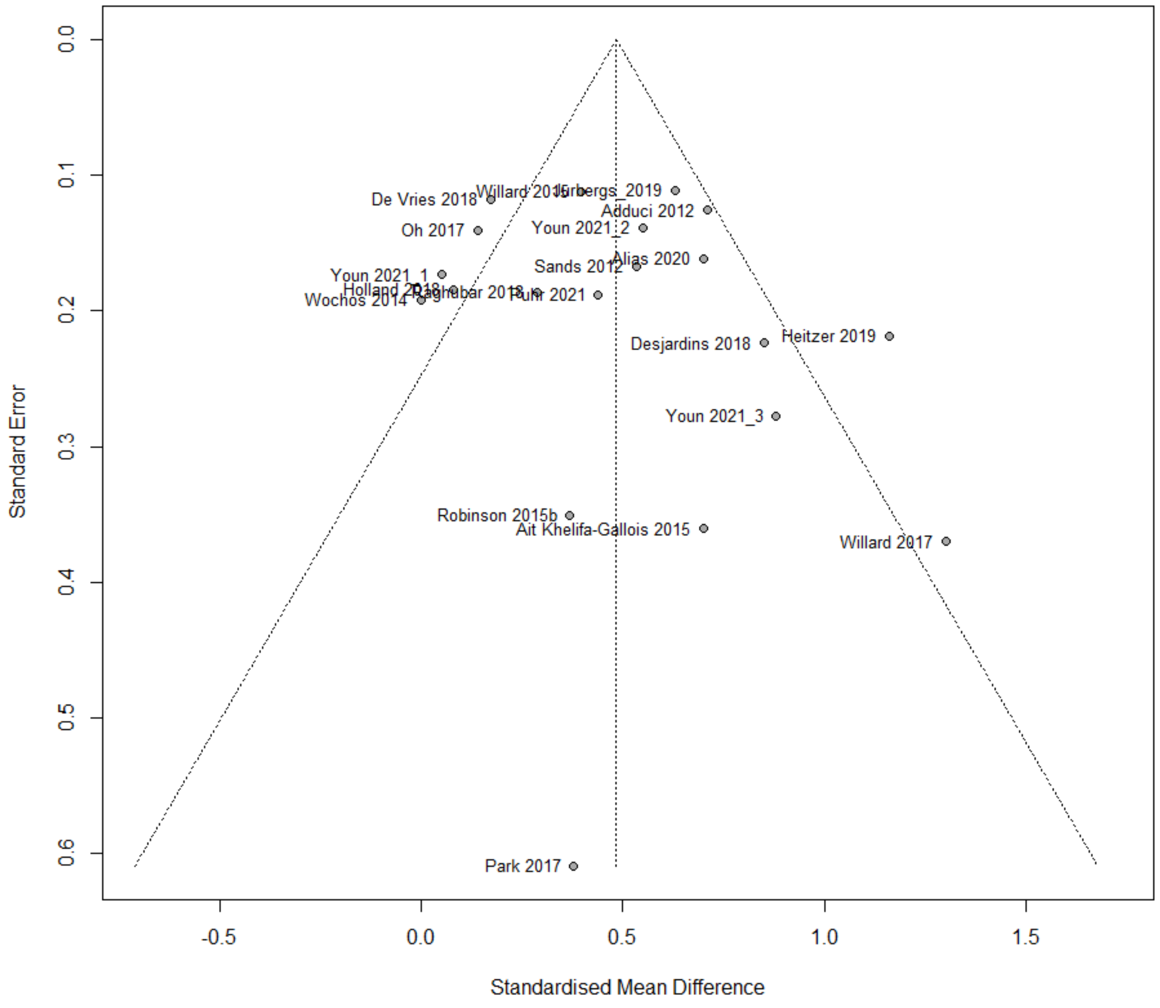

(a) Attention problems –standard mean difference

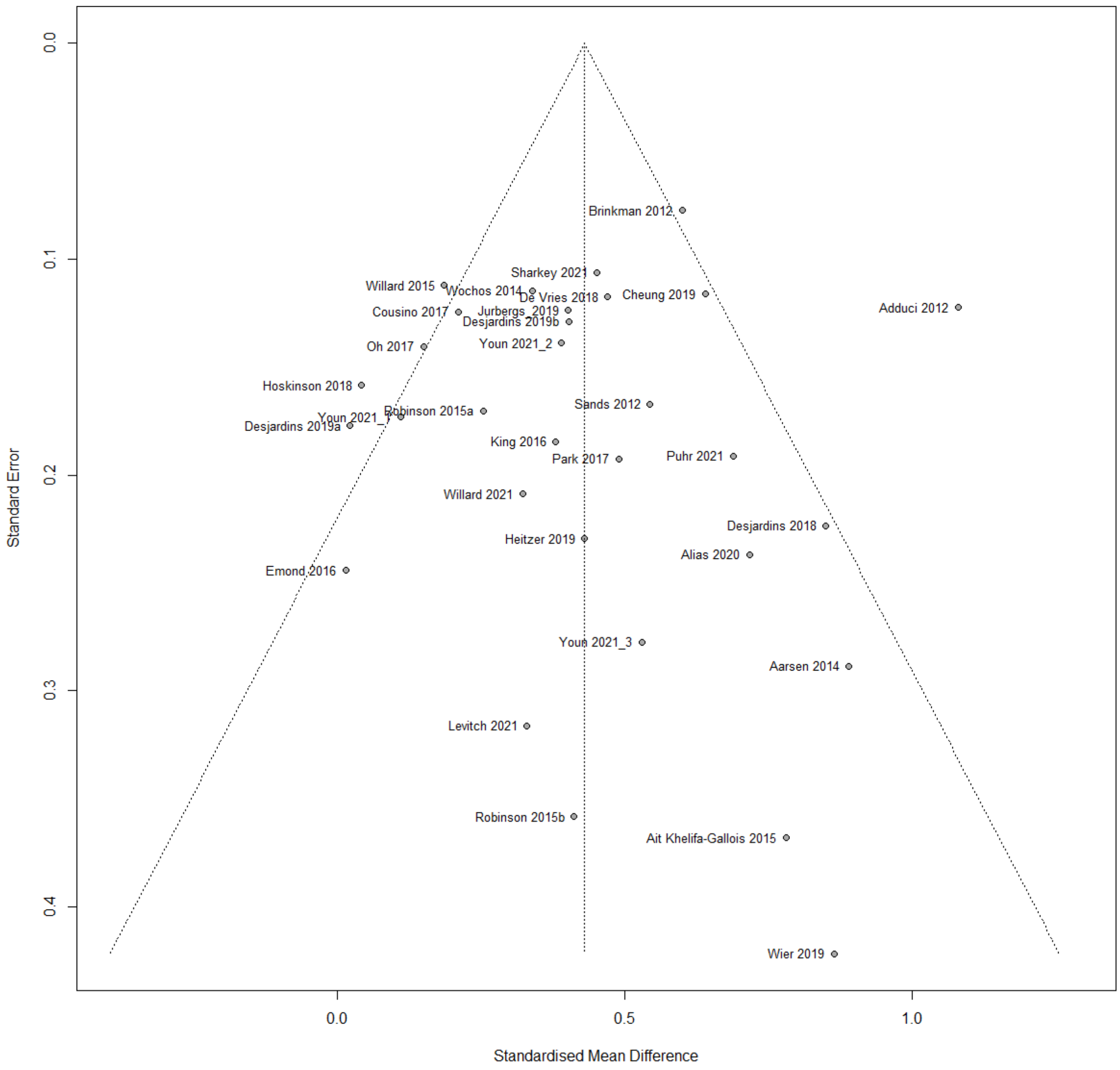

(b) Emotional difficulties –standard mean difference

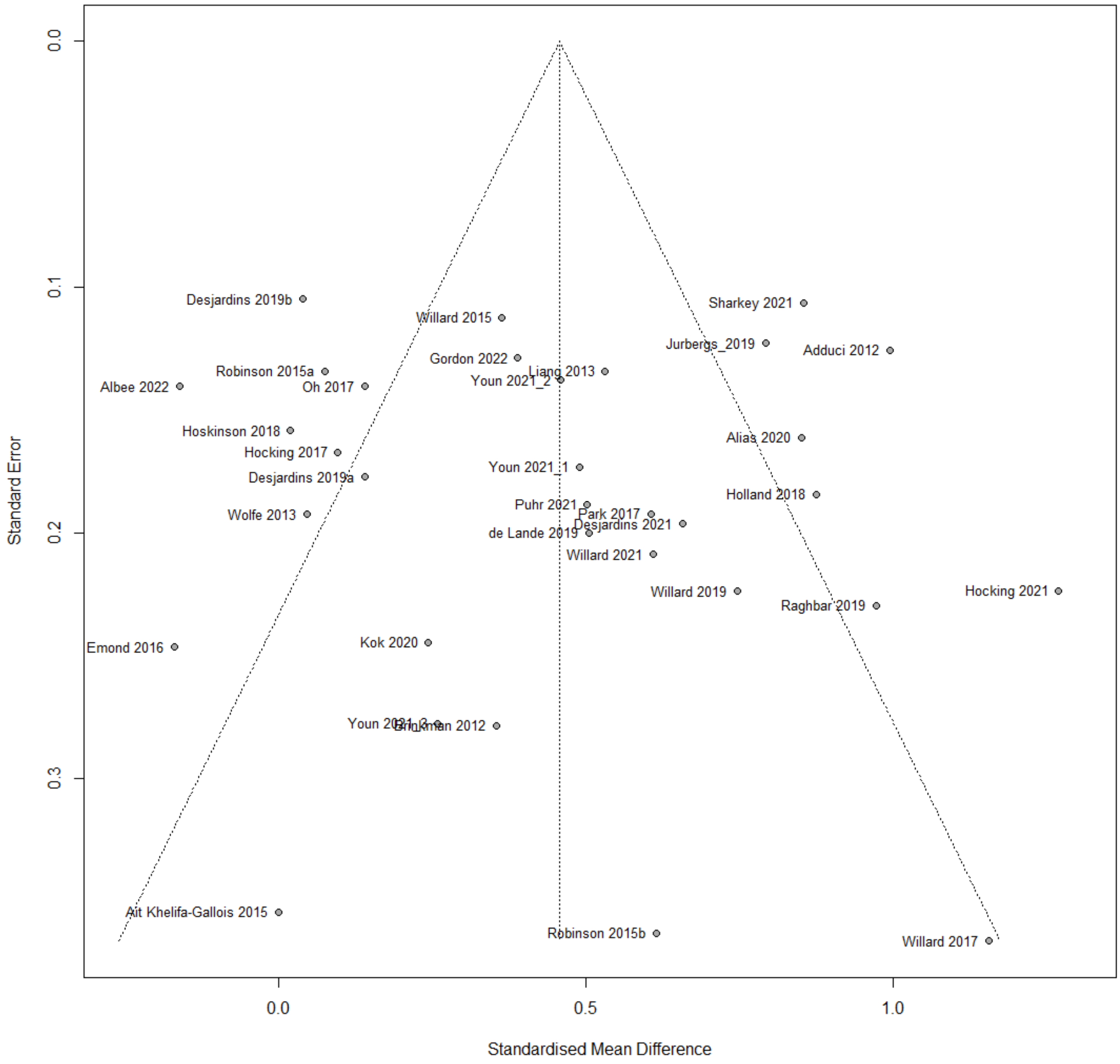

(c) Psychosocial problems –standard mean difference

Figure S6. (a-c) Funnel plot of standard mean differences of neurobehavioral impairment in paediatric brain tumor survivors compared to population norm or healthy control.

Sensitivity analysis excluding studies with high risk of bias and low sample size (n < 30).

Study	Events	Total	GLMM, Random, 95% CI
Holland 2018	1	33	0.03 [0.00; 0.16]
Youn 2021_1	0	33	0.00 [0.00; 0.11]
Dessens 2016_2	2	35	0.06 [0.01; 0.19]
Sands 2012	6	36	0.17 [0.06; 0.33]
Oh 2017	15	51	0.29 [0.17; 0.44]
Youn 2021_2	8	52	0.15 [0.07; 0.28]
Wochos 2014	5	62	0.08 [0.03; 0.18]
Adduci 2012	9	64	0.14 [0.07; 0.25]
Jurbergs_2019	18	66	0.27 [0.17; 0.40]
Willard 2015	6	80	0.07 [0.03; 0.16]
Hardy 2018	25	105	0.24 [0.16; 0.33]
Shabason 2019	81	528	0.15 [0.12; 0.19]
**Total (95% CI)**		**1145**	**0.13 [0.08; 0.19]**

Heterogeneity: $Tau^2 = 0.4265$; $Chi^2 = 29.34$, df = 11 (P < 0.01); $I^2 = 63\%$

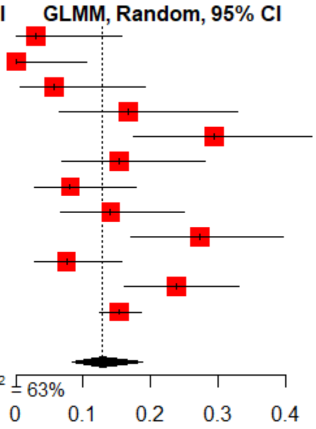

(a) Absolute risk – attention problems

Study	TE	SE	Weight	Std. Mean Difference IV, Random, 95% CI
Adduci 2012	0.05	0.1732	8.3%	0.05 [-0.29; 0.39]
Ait Khelifa-Gallois 2015	0.08	0.1844	7.9%	0.08 [-0.28; 0.44]
Park 2017	0.53	0.1670	8.5%	0.53 [0.21; 0.86]
Willard 2015	0.70	0.1612	8.7%	0.70 [0.38; 1.02]
Youn 2021_1	0.44	0.1879	7.8%	0.44 [0.07; 0.81]
Robinson 2015b	0.14	0.1404	9.5%	0.14 [-0.14; 0.41]
De Vries 2018	0.00	0.1918	7.6%	0.00 [-0.38; 0.38]
Jurbergs_2019	0.71	0.1257	10.1%	0.71 [0.46; 0.96]
Raghubar 2018	0.63	0.1112	10.6%	0.63 [0.41; 0.85]
Willard 2017	0.17	0.1175	10.4%	0.17 [-0.06; 0.40]
Alias 2020	0.40	0.1122	10.6%	0.40 [0.18; 0.62]
**Total (95% CI)**			**100.0%**	**0.36 [0.20; 0.52]**

Heterogeneity: $Tau^2 = 0.0492$; $Chi^2 = 32.99$, df = 10 (P < 0.01); $I^2 = 70\%$

Std. Mean Difference IV, Random, 95% CI

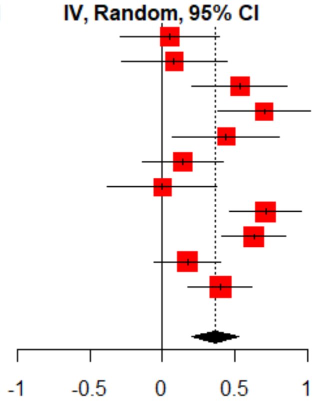

(b) Standard mean difference – attention problems

Study	Events	Total	GLMM, Random, 95% CI
Desjardins 2019a	3	32	0.09 [0.02; 0.25]
Brinkman 2012	2	33	0.06 [0.01; 0.20]
Youn 2021_1	0	33	0.00 [0.00; 0.11]
Dessens 2016_2	2	35	0.06 [0.01; 0.19]
Sands 2012	12	35	0.34 [0.19; 0.52]
Hoskinson 2018	3	40	0.07 [0.02; 0.20]
Youn 2021_2	8	52	0.15 [0.07; 0.28]
Robinson 2015a	7	56	0.12 [0.05; 0.24]
Wochos 2014	7	62	0.11 [0.05; 0.22]
Adduci 2012	41	64	0.64 [0.51; 0.76]
Cousino 2017	22	65	0.34 [0.23; 0.47]
Jurbergs_2019	14	66	0.21 [0.12; 0.33]
Willard 2015	8	80	0.10 [0.04; 0.19]
Desjardins 2019b	14	87	0.16 [0.09; 0.26]
Sharkey 2021	18	89	0.20 [0.12; 0.30]
Moitra & armstrong 2013	26	91	0.29 [0.20; 0.39]
King 2016	28	198	0.14 [0.10; 0.20]
**Total (95% CI)**		**1118**	**0.16 [0.10; 0.23]**

Heterogeneity: $Tau^2 = 0.7687$; $Chi^2 = 99.25$, df = 16 (P < 0.01); $I^2 = 84\%$

GLMM, Random, 95% CI

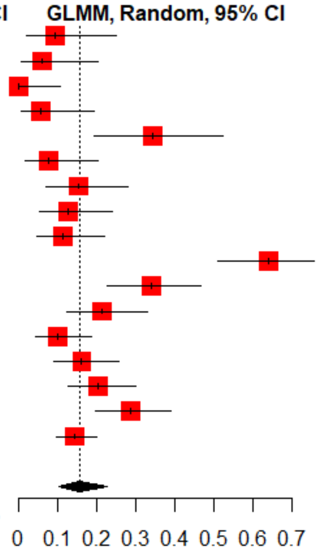

(c) Absolute risk – emotional difficulties

Study or Subgroup	TE	SE	Weight	Std. Mean Difference IV, Random, 95% CI
`Control group` = norm				
Desjardins 2019a	0.02	0.1772	4.4%	0.02 [-0.33; 0.37]
Brinkman 2012	0.60	0.0775	6.5%	0.60 [0.45; 0.75]
Youn 2021_1	0.11	0.1732	4.5%	0.11 [-0.23; 0.45]
Sands 2012	0.54	0.1670	4.6%	0.54 [0.22; 0.87]
Hoskinson 2018	0.04	0.1584	4.8%	0.04 [-0.27; 0.35]
Youn 2021_2	0.39	0.1389	5.2%	0.39 [0.12; 0.66]
Robinson 2015a	0.25	0.1703	4.5%	0.25 [-0.08; 0.59]
Adduci 2012	1.08	0.1225	5.5%	1.08 [0.84; 1.32]
Cousino 2017	0.21	0.1245	5.5%	0.21 [-0.03; 0.45]
Jurbergs_2019	0.40	0.1237	5.5%	0.40 [0.16; 0.64]
De Vries 2018	0.47	0.1175	5.6%	0.47 [0.24; 0.70]
Cheung 2019	0.64	0.1162	5.7%	0.64 [0.41; 0.87]
Willard 2015	0.19	0.1122	5.8%	0.19 [-0.03; 0.41]
Desjardins 2019b	0.40	0.1288	5.4%	0.40 [0.15; 0.65]
Sharkey 2021	0.45	0.1063	5.9%	0.45 [0.24; 0.66]
**Total (95% CI)**			**79.3%**	**0.40 [0.26; 0.54]**
Heterogeneity: $Tau^2 = 0.0558$; $Chi^2 = 60.54$, df = 14 ($P < 0.01$); $I^2 = 77\%$				
`Control group` = HC				
Emond 2016	0.01	0.2443	3.2%	0.01 [-0.46; 0.49]
Alias 2020	0.72	0.2369	3.4%	0.72 [0.25; 1.18]
Puhr 2021	0.69	0.1910	4.1%	0.69 [0.31; 1.06]
Wochos 2014	0.34	0.1146	5.7%	0.34 [0.11; 0.56]
**Total (95% CI)**			**16.4%**	**0.44 [0.14; 0.74]**
Heterogeneity: $Tau^2 = 0.0583$; $Chi^2 = 6.8$, df = 3 ($P = 0.08$); $I^2 = 56\%$				
`Control group` = Siblings				
King 2016	0.38	0.1844	4.3%	0.38 [0.02; 0.74]
**Total (95% CI)**			**100.0%**	**0.41 [0.29; 0.52]**

Heterogeneity: $Tau^2 = 0.0481$; $Chi^2 = 67.51$, df = 19 ($P < 0.01$); $I^2 = 72\%$

Test for subgroup differences: $Chi^2 = 0.08$, df = 2 ($P = 0.96$)

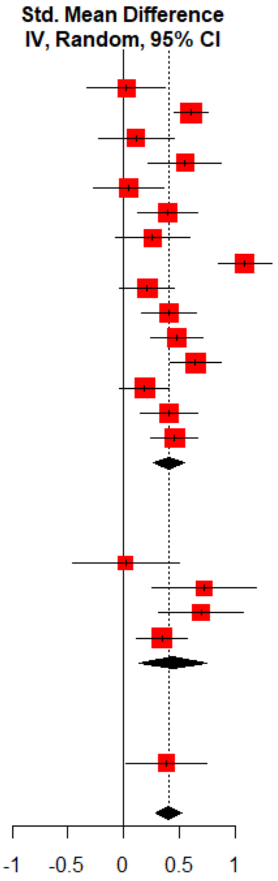

(d) Standard mean difference – emotional difficulties

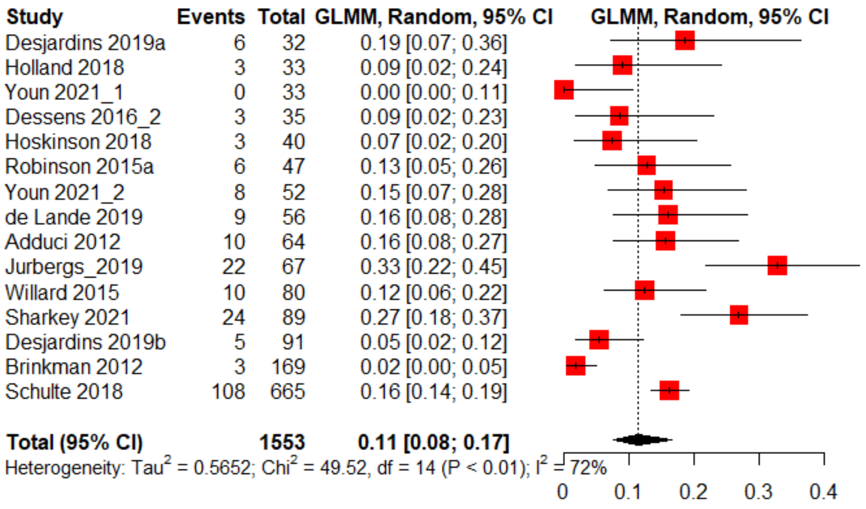

(e) Absolute risk – psychosocial problems

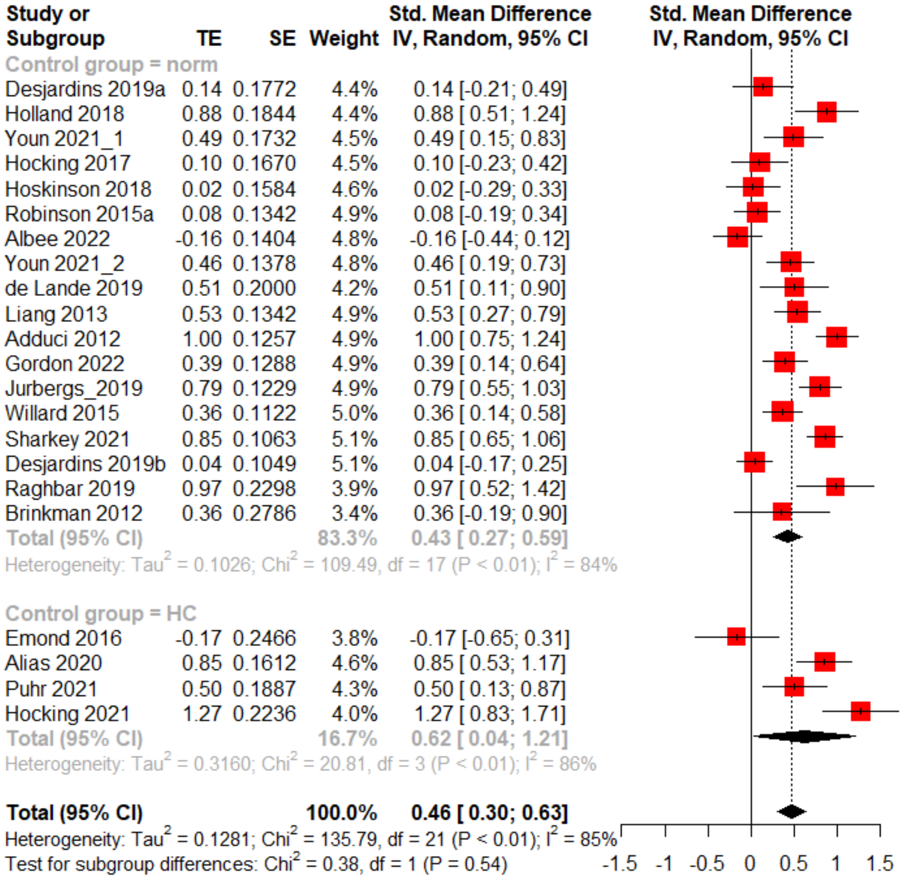

(f) Standard mean difference – psychosocial problems

Figure S7. (a-f) The absolute risk and standard mean difference (as compared to population norm or healthy controls) of pediatric brain tumor survivors having neurobehavioral impairment according to different reporting methods, excluding studies with high risk of bias and low sample size (n < 30).
