# Supplementary material for: Neurobehavioral Impairment in Pediatric Brain Tumor Survivors: A Meta-Analysis
Source: Cancers (Basel). 2022 Jul 4;14(13):3269. doi: 10.3390/cancers14133269 (PMC9265927; doi:10.3390/cancers14133269)
Supplement: Supplementary file 1 [file cancers-14-03269-s001.zip › File S1.pdf]

Embase 744 results

(("CNS tumor*" or "brain tumor*" or "brain Oncology*" or neuro-oncology* or medulloblastomas* or "pilocytic astrocytoma*" or craniopharyngiomas* or "germ cell tumors*" or glimoa* or ependymal* or glioneuronal* or embryonal*) and (children* or pediatric* or adolescent* or toddler* or preschool* or teen* or teenager* or childhood*) and ("social difficulties*" or "social outcome*" or "social problems*" or "social deficits*" or "emotional difficulties*" or "emotional problems*" or "attention deficits*" or "attention problems*" or adhd* or "attention deficit hyperactivity disorder*" or "autism spectrum disorder*" or autism* or "developmental outcomes*" or "behavioral difficulties*" or neurobehavior* or neuropsychological* or psychiatric* or psychosocial* or depression* or anxiety* or internalizing* or externalizing*)).tw
limit 1 to (human and english language and yr="2012 - 2022")

Web of science – 664 results

((TS=("CNS tumor" OR "brain tumor" OR "brain Oncology" or neuro-oncology OR medulloblastomas OR "pilocytic astrocytoma" OR craniopharyngiomas OR "germ cell tumors" OR glioma OR ependymal OR glioneuronal OR embryonal)) AND TS=(children OR pediatric OR adolescent OR toddler OR preschool OR teen OR teenager OR childhood in Title Abstract Keyword AND "social difficulties" OR "social outcome" OR "social problems" OR "social deficits")) AND TS=("social difficulties" OR "social outcome" OR "social problems" OR "social deficits" OR "emotional difficulties" OR "emotional problems" OR "attention deficits" OR "attention problems" OR adhd OR "attention deficit hyperactivity disorder" OR “autism spectrum disorder” OR autism OR "developmental outcomes" OR "behavioral difficulties" OR neurobehavior OR neuropsychological OR psychiatric OR psychosocial OR depression OR anxiety OR internalizing OR externalizing)

Scopus 979 results

(TITLE-ABS-KEY ("CNS tumor" OR "brain tumor" OR "brain Oncology" OR neuro-oncology OR medulloblastomas OR "pilocytic astrocytoma" OR craniopharyngiomas OR "germ cell tumors" OR glioma OR ependymal OR glioneuronal OR embryonal) AND TITLE-ABS-KEY (children OR pediatric OR adolescent OR toddler OR preschool OR teen OR teenager OR childhood) AND TITLE-ABS-KEY ("social difficulties" OR "social outcome" OR "social problems" OR "social deficits" OR "emotional difficulties" OR "emotional problems" OR "attention deficits" OR "attention problems" OR adhd OR "attention deficit hyperactivity disorder" OR "autism spectrum disorder" OR autism OR "developmental outcomes" OR "behavioral difficulties" OR neurobehavior OR neuropsychological OR psychiatric OR psychosocial OR depression OR anxiety OR internalizing OR externalizing)) AND (LIMIT-TO (PUBYEAR , 2022) OR LIMIT-TO (PUBYEAR , 2021) OR LIMIT-TO (PUBYEAR , 2020) OR LIMIT-TO (PUBYEAR , 2019) OR LIMIT-TO (PUBYEAR , 2018) OR LIMIT-TO (PUBYEAR , 2017) OR LIMIT-TO (PUBYEAR , 2016) OR LIMIT-TO (PUBYEAR , 2015) OR LIMIT-TO (PUBYEAR , 2014) OR LIMIT-TO (PUBYEAR , 2013) OR LIMIT-TO (PUBYEAR , 2012)) AND (LIMIT-TO (DOCTYPE , "ar")) AND (LIMIT-TO (LANGUAGE , "English"))

Cochrane 209 results

"CNS tumor" OR "brain tumor" OR "brain Oncology" or neuro-oncology OR medulloblastomas OR "pilocytic astrocytoma" OR craniopharyngiomas OR "germ cell tumors" OR glioma OR ependymal OR glioneuronal OR embryonal in Title Abstract Keyword AND children OR pediatric OR adolescent OR toddler OR preschool OR teen OR teenager OR childhood in Title Abstract Keyword AND "social difficulties" OR "social outcome" OR "social problems" OR "social deficits" OR "emotional difficulties" OR "emotional problems" OR "attention deficits" OR "attention problems" OR adhd OR "attention deficit hyperactivity disorder" OR “autism spectrum disorder” OR autism OR "developmental outcomes" OR "behavioral difficulties" OR neurobehavior OR neuropsychological OR psychiatric OR psychosocial OR depression OR anxiety OR internalizing OR externalizing in Title Abstract Keyword - (Word variations have been searched)

PubMed 764 results

("CNS tumor"[Text Word] OR "brain tumor"[Text Word] OR "brain Oncology"[Text Word] OR neuro-oncology [Text Word] OR medulloblastomas[Text Word] OR "pilocytic astrocytoma"[Text Word] OR craniopharyngiomas[Text Word] OR "germ cell tumors"[Text Word] OR glioma[Text Word] OR ependymal[Text Word] OR glioneuronal[Text Word] OR embryonal[Text Word]) AND (children[Text Word] OR pediatric[Text Word] OR adolescent[Text Word] OR toddler[Text Word] OR preschool[Text Word] OR teen[Text Word] OR teenager[Text Word] OR childhood[Text Word])) AND ("social difficulties"[Text Word] OR "social outcome"[Text Word] OR "social problems"[Text Word] OR "social deficits"[Text Word] OR "emotional difficulties"[Text Word] OR "emotional problems"[Text Word] OR "attention deficits"[Text Word] OR "attention problems"[Text Word] OR adhd[Text Word] OR "attention deficit hyperactivity disorder"[Text Word] OR "autism spectrum disorder" [Text Word] OR autism[Text Word] OR "developmental outcomes"[Text Word] OR "behavioral difficulties"[Text Word] OR neurobehavior[Text Word] OR neuropsychological[Text Word] OR psychiatric[Text Word] OR psychosocial[Text Word] OR depression[Text Word] OR anxiety[Text Word] OR internalizing[Text Word] OR externalizing[Text Word])
