# Supplementary material for: Neurobehavioral Impairment in Pediatric Brain Tumor Survivors: A Meta-Analysis
Source: Cancers (Basel). 2022 Jul 4;14(13):3269. doi: 10.3390/cancers14133269 (PMC9265927; doi:10.3390/cancers14133269)
Supplement: Supplementary file 1 [file cancers-14-03269-s001.zip › File S2.pdf]

Assessment scale	Report method (Used in the reviewed papers) [1]	Frequency in 50 reviewed studies
CBCL: Child Behavior Checklist [96]	Parent-report	22
ABAS-II: Adaptive Behavior Assessment System-Second Edition [97]	Parent-report	5
BRIEF: Behavior Ratings of Executive Function [98]	Parent-report	5
BASC: Behavior Assessment System for Children [99]	Parent-report	5
SSIS : Psychosocial Skills Improvement System [100]	Parent-report/self-report	4
YSR: Youth Self-Report [101]	Self-report	3
VABS: Vineland Adaptive Behavior Scales [102]	Parent-report	2
SDQ: Strengths and Difficulties Questionnaire [103]	Parent-report	2
NTEM: NIH Toolbox—Emotion Measures [104]	Parent-report	2
SSRS: Psychosocial Skills Rating System [105]	Parent-report	2
SRS: The Psychosocial Responsiveness Scale [106]	Parent-report/ teacher-report	2
CES-DC: Center for Epidemiological Studies Depression Scale for Children [107]	Self-report	1
SPPC: Self-Perception Profile for Children [108]	Self-report	1
ADHD-RS-IV: ADHD Rating Scale-IV [109]	Parent-report/ teacher-report	1
BSI: Brief Symptom Inventory–18 [110]	Self-report	1
BAI: Beck Anxiety Inventory [111]	Self-report	1

[1]Some scales have both self-report and parent-report forms, here we list the report form used in the reviewed papers.

BDI: Beck Depression Inventory [112]	Self-report	1
SCARED–C: Screen for Child Anxiety Related Emotional Disorders–Child version [113]	Self-report	1
BYI: Beck Youth Inventories [114]	Self-report	1
K-PRC: Korean Personality Rating scale for Children [115]	Parent-report	1
NFI: Neuropsychological Functioning Inventory [116]	Parent-report/ Self-report	1
SEARS: Psychosocial-Emotional Assets and Resilience Scales [117]	Parent-report/ Self-report/ Teacher-report	1

96. Achenbach, T.M. and C. Edelbrock, Child behavior checklist. *Burlington (Vt)* **1991**. *7*, 371–392.
97. Gray, S.A.O. and A.S. Carter, *Adaptive Behavior Assessment System,* Second Edition, in Encyclopedia of Autism Spectrum Disorders, F.R. Volkmar, Editor; Springer New York: New York, NY, USA, 2013; 52–55.
98. Roth, R.M.; Gioia, G.A.; Guy, S.C.; Kenworthy, L.; Isquith, P.K. *Behavior Rating Inventory of Executive Function: BRIEF.*; Psychological Assessment Resources: Odessa, FL, USA, 2000.
99. Reynolds, C.R., Behavior assessment system for children. *The Corsini encyclopedia of psychology*, **2010**, 1–2. https://doi.org/10.1002/9780470479216.corpsy0114
100. Gresham, F.M. and S.N. Elliott, *Social skills improvement system: Rating scales manual*. NCS Pearson: Bloomington, MN, USA, 2008.
101. Achenbach, T.M. *Manual for the Youth Self-Report and 1991 Profile*; Department of Psychiatry, University of Vermont: Burlington, VT, USA, 1991.
102. Sparrow, S.; Cicchetti, D.; Saulnier, C. *Vineland Adaptive Behavior Scales*—3rd Edition; Pearson: Bloomington, MN, USA, 2016.
103. Muris, P., C. Meesters, and F. van den Berg, The strengths and difficulties questionnaire (SDQ). *European child & adolescent psychiatry* **2003**. *12*, 1–8.
104. Gershon, R.C.; Cella, D.; A Fox, N.; Havlik, R.J.; Hendrie, H.C.; Wagster, M.V. Assessment of neurological and behavioural function: the NIH Toolbox. *Lancet Neurol.* **2010**, *9*, 138–139, https://doi.org/10.1016/s1474-4422(09)70335-7.
105. Gresham, F.M.; Elliott, S.N.; Service, A.G. *Social Skills Rating System Manual*; American Guidance Service: Circle Pines, MN, USA, 1990.
106. Constantino, J.N.; Gruber, C. *Social Responsiveness Scale (SRS)*; Western Psychological Services: Los Angeles, CA, USA, 2005.
107. Shahid, A.; Wilkinson, K.; Marcu, S.; Shapiro, C.M. Center for Epidemiological Studies Depression Scale for Children (CES-DC). **2011**, 93–96, https://doi.org/10.1007/978-1-4419-9893-4_16.
108. Harter, S., *Self-perception profile for children: manual and questionnaires (grades 3–8)*. University of Denver: Denver, CO, USA, 2012.
109. Pappas, D., ADHD Rating Scale-IV: Checklists, norms, and clinical interpretation. *Journal of psychoeducational assessment* **2006**, *24*, 172–178.
110. Derogatis, L. *BSI-18: Brief Symptom Inventory 18—Administration, Scoring, and Procedures Manual*; NCS Pearson: Minneapolis, MN, USA, 2001.

111. Beck, A.T.; Epstein, N.; Brown, G.; Steer, R. *Beck Anxiety Inventory Manual*; Psychological Corp: San Antonia, TX, USA, 1993.
112. Beck, A.T., R.A. Steer, and G.K. Brown, Beck depression inventory; Harcourt Brace Jovanovich: New York, NY, USA, 1987.
113. Monga, S., et al., Screen for child anxiety-related emotional disorders (SCARED): Convergent and divergent validity. *Depression and anxiety*, **2000**, *12*, 85–91.
114. Beck, J.S.; Beck, A.T.; Jolly, J.B. *Beck Youth Inventories of Emotional & Social Impairment: Depression Inventory for Youth, Anxiety Inventory for Youth, Anger Inventory for Youth, Disruptive Behavior for Youth, Self-Concept Inventory for Youth: Manual*; Psychological Corporation: New York, NY, USA, 2001.
115. Cho, S. A standardization study of the Korean Personality Rating Scale for Children (KPRC). *Korean J. Clin. Psychol*. **2006**, *25*, 825–848.
116. Sandberg, M.A., *Neurobehavioral Functioning Inventory, in Encyclopedia of Clinical Neuropsychology;* Springer International Publishing: Cham, Switzerland, 2018, 2383-2384.
117. Torki, R.N.; Doerner, E.E.; Kaye, N.C.; Tsutsui, E.M.; Merrell, K.W. Social Emotional Assets and Resilience Scales: Status and New Developments. **2010**, https://doi.org/10.1037/e618172010-001.
